# On the evolution and development of morphological complexity: A view from gene regulatory networks

**DOI:** 10.1371/journal.pcbi.1008570

**Published:** 2021-02-24

**Authors:** Pascal F. Hagolani, Roland Zimm, Renske Vroomans, Isaac Salazar-Ciudad

**Affiliations:** 1 Evo-devo Helsinki community, Centre of Excellence in Experimental and Computational Developmental Biology, Institute of Biotechnology, University of Helsinki, Helsinki, Finland; 2 Institute of Functional Genomics, École Normale Superieure, Lyon, France; 3 Konrad Lorenz Insititute for Evolution and Cognition Research, Vienna, Austria; 4 Origins Center, Nijenborgh, Groningen, The Netherlands; 5 Informatics Institute, University of Amsterdam, Amsterdam, The Netherlands; 6 Genomics, Bioinformatics and Evolution group, Departament de Genètica i Microbiologia, Universitat Autònoma de Barcelona, Cerdanyola del Vallès, Spain; 7 Centre de Rercerca Matemàtica, Cerdanyola del Vallès, Spain; OvGU; Medical Faculty, GERMANY

## Abstract

How does morphological complexity evolve? This study suggests that the likelihood of mutations increasing phenotypic complexity becomes smaller when the phenotype itself is complex. In addition, the complexity of the genotype-phenotype map (GPM) also increases with the phenotypic complexity. We show that complex GPMs and the above mutational asymmetry are inevitable consequences of how genes need to be wired in order to build complex and robust phenotypes during development.

We randomly wired genes and cell behaviors into networks in EmbryoMaker. EmbryoMaker is a mathematical model of development that can simulate any gene network, all animal cell behaviors (division, adhesion, apoptosis, etc.), cell signaling, cell and tissues biophysics, and the regulation of those behaviors by gene products. Through EmbryoMaker we simulated how each random network regulates development and the resulting morphology (i.e. a specific distribution of cells and gene expression in 3D). This way we obtained a zoo of possible 3D morphologies. Real gene networks are not random, but a random search allows a relatively unbiased exploration of what is needed to develop complex robust morphologies. Compared to the networks leading to simple morphologies, the networks leading to complex morphologies have the following in common: 1) They are rarer; 2) They need to be finely tuned; 3) Mutations in them tend to decrease morphological complexity; 4) They are less robust to noise; and 5) They have more complex GPMs. These results imply that, when complexity evolves, it does so at a progressively decreasing rate over generations. This is because as morphological complexity increases, the likelihood of mutations increasing complexity decreases, morphologies become less robust to noise, and the GPM becomes more complex. We find some properties in common, but also some important differences, with non-developmental GPM models (e.g. RNA, protein and gene networks in single cells).

## Author summary

In this study we research the space of possible networks of gene and cell interactions in development using a computational model. This model includes gene networks, cell behaviors (such as cell division, apoptosis, adhesion, etc.) and realistic cell biophysics, which allows us to fully simulate animal development. We found that networks leading to complex morphologies have some things in common, which distinguish them from the networks leading to simple morphologies: 1) They are rarer; 2) They need to be finely tuned; 3) Mutations tend to decrease morphological complexity; 4) They are less robust; and 5) They have more complex genotype-phenotype maps. These results imply that, when complexity evolves, it does so at a progressively slower rate as mutations increasing complexity become rarer, morphologies become less robust to noise, and more complex genotype-phenotype maps evolve.

## Introduction

There is no consensus for the definition of complexity, yet one of the most salient characteristics of living beings is their complexity. Explaining such complexity is one of the fundamental questions of biology. In every generation, development generates complexity starting from a simple initial condition, e.g. a zygote. In addition, between generations, due to evolution, morphological complexity can also change. There is a clear relationship between development and morphological evolution; any change in morphology during evolution is first a change in development.

**Fig 1:**
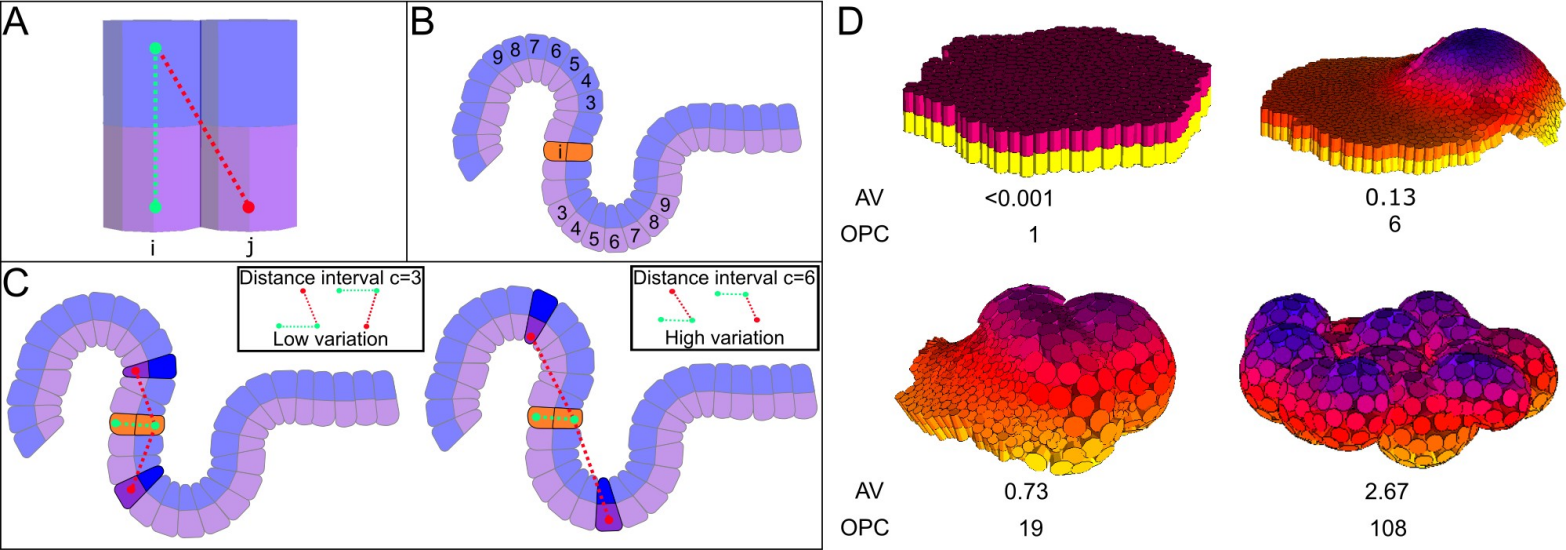
Angle variation (AV): method to measure the complexity of an epithelium. The AV measure of complexity is based on the variance of the angles between epithelial cells [10]. (A) The angle between two cells *i* and *j* is calculated as the angle between two vectors, the vector between the apical and basal node of epithelial cell *i* (green dotted lines) and the vector between the basal node of cell *j* and the apical node of cell *i* (red dotted line). (B) For each cell *i* we calculate the angles to all other epithelial cells. Each cell *j* is grouped into a distance category based on its distance to cell *i* (there are six of these distance categories). Each category includes the nodes at a given distance interval to *i*, defined as follows: $D_{c}=\{c\times p^{\overset{´}{ADD}},(c+1)+p^{\overset{´}{ADD}}\};c\in \{3,\ldots ,9\}$ Where *D*_*c*_ is the distance interval in which cell *j* has to fall in order to be included in the category *c*. *c* defines the range of each interval. $p^{\overset{´}{ADD}}$ is the average distance of adhesion (S1 Text) of all epithelial cells in the whole embryo. This is a measure of the average cell size. The minimal *c* we use is 3. The largest value is *9*. This value allows to consider the macro-structure of the embryo (i.e. large-scale morphological complexity). (C) For each category we calculate the variance and sum them together. This way, the angle variation complexity (AV) is: $AV=\frac{\sum_{i=1}^{n}\sum_{c=3}^{9}V_{ic}}{7n}$ Where *i* is each of the epithelial cells, *n* is the number of epithelial cells in the embryo, *c* is each of the categories intervals and *V*_*ic*_ is the angle variation for cell *i* in the category *c*. (D) Shows examples of morphologies found in the ensemble and their complexity. The complexity of the morphologies is shown in both the Angle Variation (AV) and the Orientation Patch Count (OPC) morphological complexity measures (see S1A Text, Methods 4.1 and [11] for details on OPC). Color indicates the position of the nodes in the z-axis.

Complexity is a commonly used term and there may not be a unique, clear-cut, and quantifiable definition [1]. There is, instead, a diversity of definitions, each likely to elicit criticisms by defenders of other definitions or by those that consider that a definition is not possible or worthwhile [2–9]. Different definitions may also be more suitable for different aspects of nature without any definition being necessarily superior to the rest. In the case of 3D morphology, for example, one can focus on whether its constituting cells are distributed in a regular predictable way, such as in a flat sheet or a sphere; or not, as in a crumpled paper. Herein, we take this approach and define complexity based on how difficult it is to guess the 3D coordinates of a cell based on the positions of its immediate neighbors (see Fig 1, S1A Text and Methods 4).

The difficulty in defining and measuring morphological complexity have not precluded studying its evolution. Some studies argue that there is a passive trend of increasing complexity in evolution [12–18], while others argue that the trend is due to natural selection [7,19–22]. Yet, other studies argue that there is not such a trend or at least it is not currently detectable [23,24]. In this study we do not assume or propose that there are trends in the evolution of complexity. Instead, we want to understand how complexity evolves when it does, i.e. in those lineages where it increased.

Complex morphologies evolved from simpler morphologies. Therefore, to understand the evolution of complexity, we will ask how often mutations increase morphological complexity when the morphology is simple and how often they do it when morphology is already complex. In this line of thinking, recent work on a specific organ (teeth) suggests there is a mutational asymmetry. It is easier to experimentally manipulate development to produce a decrease in morphological complexity than to produce an increase [25,26]. Similar views have been expressed for natural variation in populations [27]. In this article we use a general mathematical model of embryonic development to explore whether this mutational asymmetry is a consequence, or a side-effect, of how genes and cells need to be wired into networks to produce complex robust morphologies. We will also ask if another side-effect of complex robust morphologies is to have a complex relationship between genetic and phenotypic variation, or genotype-phenotype map (GPM). The GPM is just an association, or map. The GPM does not tell which morphologies are possible or how they form in development. The GPM only shows which of the morphologies that are possible through development are associated with which specific genetic variants.

One of the major tenets of developmental evolutionary biology or evo-devo is that the GPM is very complex [28–35]. Morphology is constructed during development through complex networks of interactions between gene products, cells, and biophysical processes. In other words, genes do not have intrinsic effects on morphology. Genes affect morphology because they affect the dynamics of the networks of gene, cell and biophysics where they are embedded. In other words, the GPM does not depend only on genes or gene interactions, it also depends on cell interactions and biophysics. In this article, as elsewhere [30,36], a GPM is considered to be complex when small genetic changes can lead to relatively large morphological changes. Complex GPMs have been suggested to hamper evolution [20,37,38]: With a complex GPM, small genetic changes can lead to relatively large morphological changes and, on average, the similarity between parents and offspring decreases. In that case adaptive phenotypic variations in the parents are less likely to pass to their offspring, thus hampering evolution [20,37,38]. Studying how the complexity of the GPM differs between simple and complex morphologies is thus relevant to understand how complex morphologies can arise and evolve.

There are many theoretical studies on the general properties of the GPM. Most of these studies do not consider morphology and development, rather the other phenotypic levels and the processes (other than development) by which those levels are constructed. The mapping between the primary (genotype) and secondary (phenotype) structures of RNA can be considered a GPM that has been extensively studied by mathematical modeling [39–43]. Similar, but coarser model studies exist for protein structure [44–46] and gene networks within single cells [47–50]. Only few GPM studies consider genetic and mechanical interactions between cells in a spatial context and how these lead to complex multicellular phenotypes (i.e. morphology), as in development. The studies that do, only consider specific organs [51–56] or only consider gene networks and cell signaling [57–62] without mechanical interactions or cell behaviors. Herein, we complement these latter approaches by using a general modeling framework for development, EmbryoMaker [63], which considers gene networks and cell signaling, but also mechanical interactions and cell behaviors. This modeling approach is not specific to any organ. Instead, it considers animal development in general. The study of this model should inform us about properties of the GPM that are shared between phenotypic levels (RNA, protein, single cell gene networks, and morphology) and highlight the properties not shared between these levels.

Despite the striking complexity of animals, some maintain that their development can be accomplished by a finite number of cell behaviors and interactions [64–67]. These cell behaviors are cell division, cell adhesion, cell death, cell growth, cell contraction, extracellular matrix secretion, signaling and extracellular signal reception and cell differentiation [64–67]. Cell migration and cell shape changes that result from certain patterns of cell contraction and adhesion could also be considered as cell behaviors.

In addition to cell behaviors, development involves gene product and cell interactions. Gene products interact in networks to regulate each other, cell behaviors, and cell interactions [68]. Cell interactions during development occur mainly through molecular signals (extracellularly diffusing or membrane-bound) and forces. Signaling often involve extracellularly diffusive molecules [68], while forces are generated by cell behaviors (e.g. cell contraction). Both signaling and forces can lead to changes in the gene expression [68]. Altering gene expression can result in regulatory changes in the behaviors and the biophysical properties of cells. The gene expression in certain cells is also regulated by cell behaviors, since cell behaviors can induce cell movements, which alter the location of cells in space. This cell movements will influence the spatial allocation of extracellular signals that elicit gene expression changes in cells [64].

**Fig 2:**
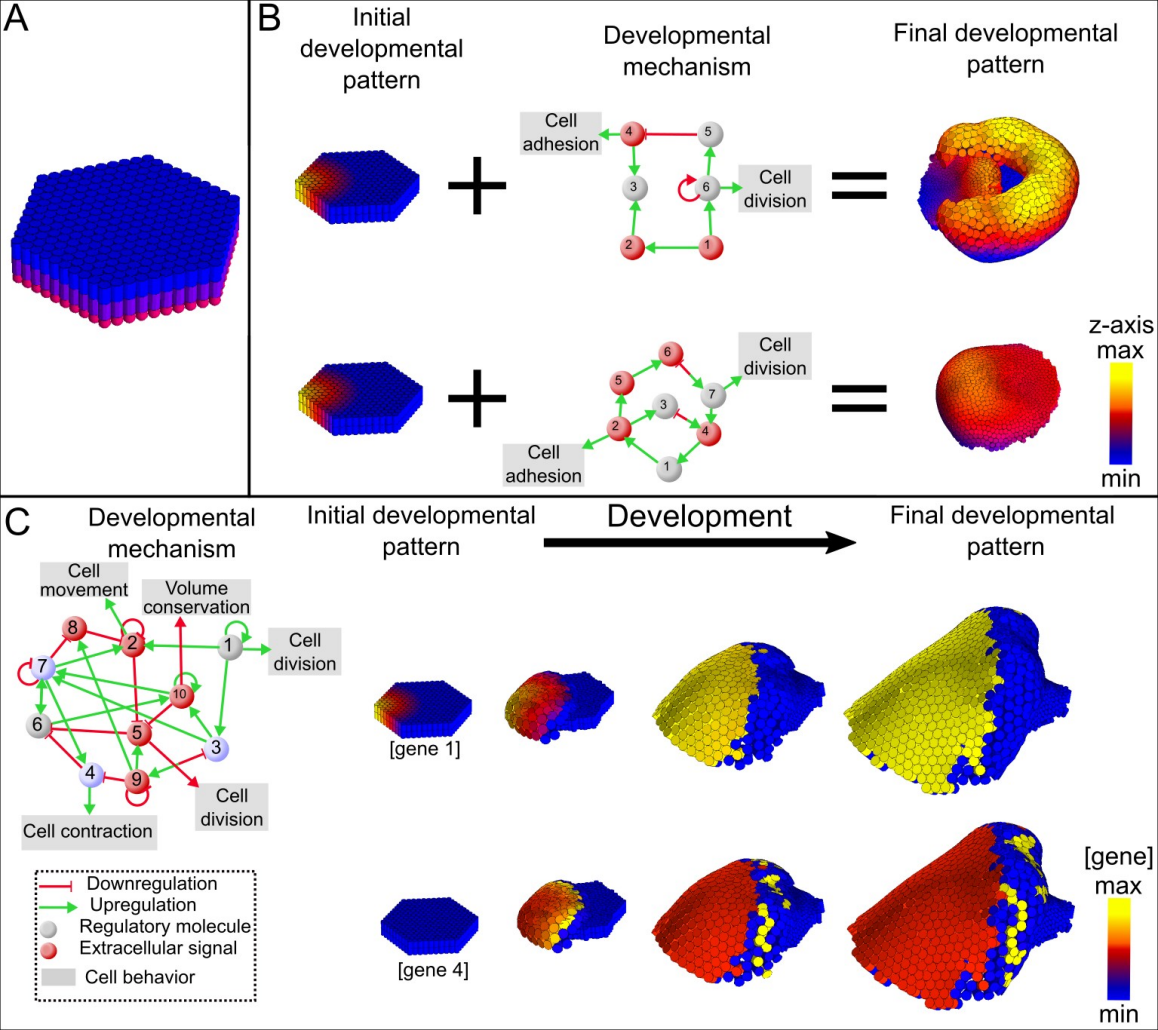
Example developmental mechanisms and pattern transformations. (A) Initial condition used in every simulation. Cylinders represent epithelial cells, the apical side in blue and the basal side in purple. The pink spheres represent mesenchymal cells. (B) The network diagrams in the center represent two idealized developmental mechanisms. Together with the initial condition depicted in their left they lead to the transformation of the initial morphology into other morphologies, on the right. In the initial morphology, color shows the starting level of expression of gene 1. In contrast, in the resulting morphology color shows the position along the z-axis. (C) One of the developmental mechanism found in this research (left). We show four different pattern transformations time points for two different gene products. The morphological and gene expression changes result from the dynamics of the developmental mechanism (left).

As in [64], we use the concept of developmental mechanism. A developmental mechanism is defined as a gene network, the cell behaviors, and cell bio-physical properties it regulates. Developmental mechanisms can be seen as necessary for transforming morphologies over developmental time, i.e. transforming a distribution of cells in 3D space. The building of morphological complexity can be seen as a sequence of such morphological transformations over developmental time (Fig 2). In turn, morphological evolution can be seen as changes in these transformations between generations.

If as suggested above, morphological transformations in development involve gene networks and a finite number of cell behaviors and cell interactions, then any computational model that includes them could potentially reproduce the range of morphological transformations possible in animal development. In this work, we used one such model, EmbryoMaker [63] to perform an ensemble study. We randomly wired gene products, cell behaviors, and cell mechanical properties into a huge number of developmental mechanisms (i.e. networks) and simulated them using EmbryoMaker to obtain a large set of morphologies, what we call the *ensemble* (see Methods 1 and S1 Text). We explored whether mutational asymmetry and complex GPMs are a general property of most or all, of the developmental mechanisms that are able to produce complex morphologies in such an ensemble.

We are fully aware that developmental mechanisms found in nature are not completely random, but the outcome of evolution. Nevertheless, a random sample of developmental mechanisms provides a general view on possible developmental mechanisms that is not restricted to those that have actually occurred in nature or to those that happen to be known to current developmental biology. In other words, by modeling a large number of different in silico morphologies and their statistical analysis we aim at understanding general principles of development and the GPM.

**Fig 3:**
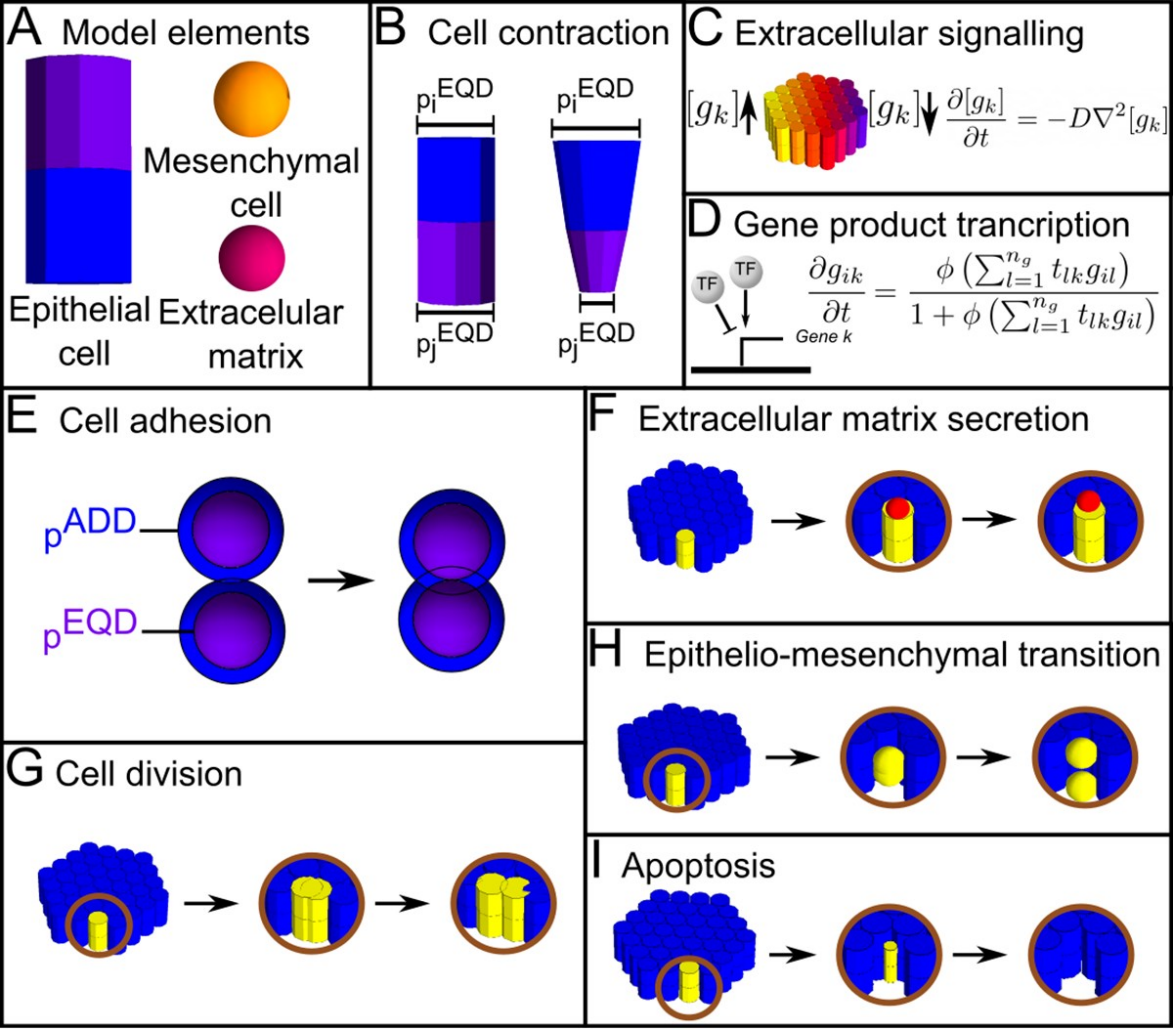
EmbryoMaker. (A) EmbryoMaker models three types of elements, epithelial and mesenchymal cells, and extracellular nodes. There are two types of epithelial nodes, apical (in violet) and basal (blue), which together form a cylinder or epithelial cell. Mesenchymal cells and extracellular matrix components are made of single spherical nodes. (B) Cell contraction. Nodes of a cell can change their size by decreasing or increasing their equilibrium radius (*p*^*EQD*^). This can result in epithelial cells with a conical shape. (C) Extracellular signaling. In order to model diffusion, the movement of molecules between nodes is implemented using Fick’s second law of diffusion. *g*_*k*_ is the concentration of gene product *k* in a node (a model variable). (D) Gene product transcription regulates the expression of a gene. *g*_*ik*_ is the amount of gene product *k* in node *i* and each *t*_*lk*_ (a model parameter) is the strength by which each specific gene product *l* activates or inhibits the gene product *k*. (E-J) Cell behaviors implemented in EmbryoMaker. (E) Cell adhesion. Two cells whose radius of adhesion (*p*^*ADD*^, blue sphere) overlap, are considered to be in contact. If they are in contact and if they adhere to each other, they will come closer together until they reach their radius of equilibrium (*p*^*EQD*^, purple sphere). (F) Extracellular matrix secretion. ECM can be secreted by any cell that expresses a gene product regulating this cell behavior. (G) Cell division. (H) Epithelial-mesenchymal transition (EMT). (I) Apoptosis. Cell undergoing apoptosis will gradually decrease their size until they reach a minimal size and are completely eliminated.

**Fig 4:**
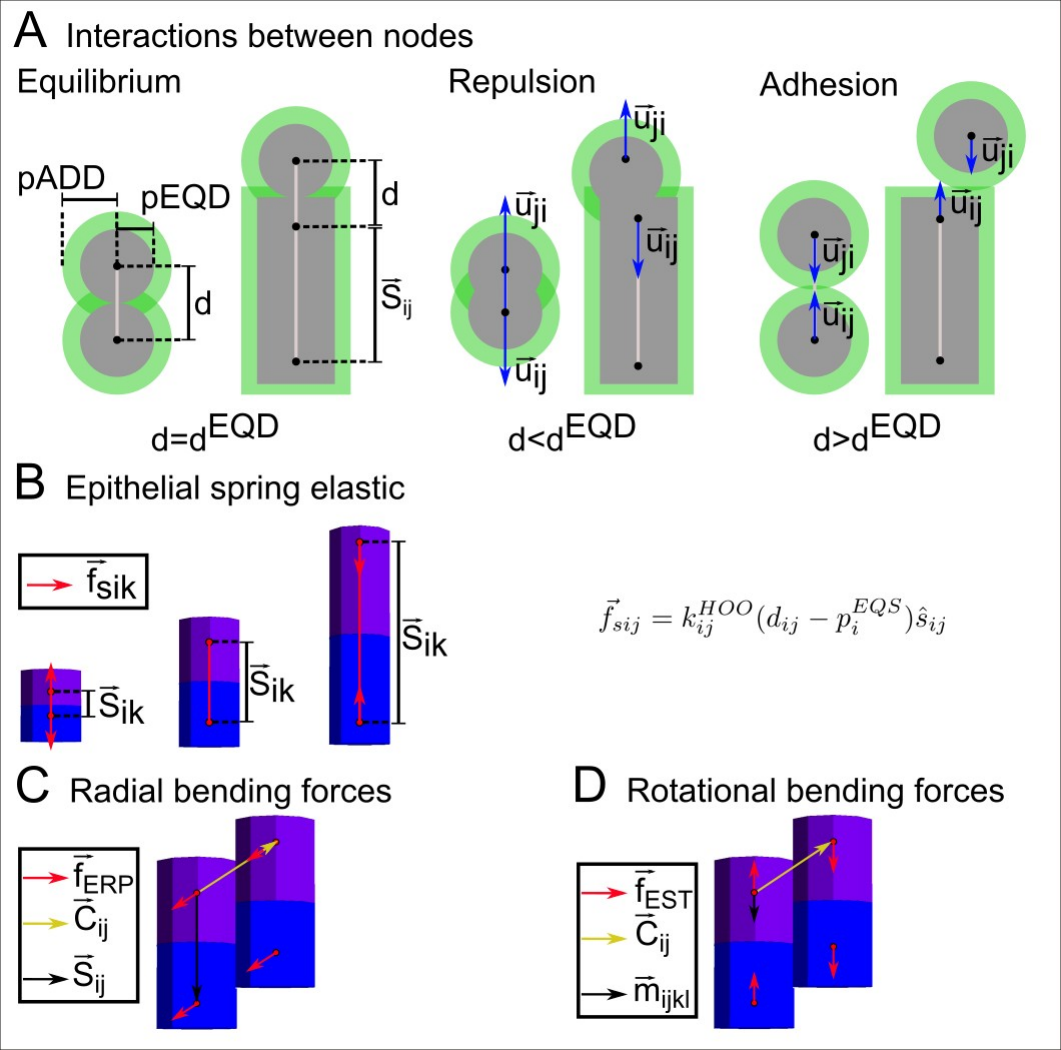
Basic mechanical interactions in EmbryoMaker. (A) When two nodes are at a distance (d) smaller than *d*^*ADD*^ they experience an attractive force, when they are closer than *d*^*EQD*^ they experience a repulsive force. *d*^*EQD*^ is simply the sum of the sizes or radius of repulsion, *p*^*EQD*^, of the nodes interacting while *d*^*ADD*^ is the sum of the radius of adhesion of the nodes interacting, *p*^*ADD*^. The values of these radii can change over time as a result of gene expression or external pressures. The direction of the interaction is from the center of one node to the center of the other (red arrows). The interaction of a spherical node (either mesenchymal or from the ECM) with the apical or basal side of an epithelial node is always parallel to the apical-basal axis of the cylinder (and perpendicular to it when the interaction is lateral). (B-D) Depict mechanical forces specific for epithelia. (B) The two nodes composing a cylinder are connected by an unbreakable spring (black line). Elastic forces will always follow the direction of that spring. The spring has an equilibrium distance (d^EQS^), if the distance between the centers of the nodes (d) in a epithelial cylinder are closer than d^EQS^ (left figure in B), elastic forces will repulse the nodes (red arrows). If the distance between the centers of the nodes is greater than d^EQS^, elastic forces will attract them (this distance is again the sum of the mechanical property, *p*^*EQS*^, of the two nodes that is itself a variable of the model). (C) Epithelial bending forces tend to put two cylinders in a position in which the angle between the vector connecting the two apical (or basal) nodes and the apical-basal axis is π/2. Epithelial bending forces apply on a direction normal to the apical/basal surface (see supplementary). (D) The bending rotational force applies in the direction connecting the two epithelial nodes from the same side (see supplementary).

EmbryoMaker is a general computational model of animal development that can simulate any gene network, most animal cell behaviors (division, adhesion, apoptosis, etc.), cell signaling, mechanical properties (see Figs 3 and 4 and S1T Text), and their interrelationships. Each cell has a number of variables that include its position in 3D space, its size, many mechanical properties, and the level of expression of genes. These variables take continuous values and change according to a set of differential equations (one equation per variable in each cell, see S1 Text, and Fig 3). Some of these equations calculate how the expression of a gene in a cell changes due to the other genes expressed in a cell and incoming extracellular signals (see Fig 3C and 3D). Secretion and diffusion of extracellular signals are calculated by other differential equations. Additional differential equations calculate how cell positions change due to pushing and pulling by neighboring cells at a given moment, and how movement is affected by the mechanical properties of each cell (see Fig 4). Similarly, a set of differential equations are used to calculate how the mechanical properties of cells change due gene regulation and incoming forces. In addition to variables (that can change over developmental time), each equation takes a set of parameters that do not change over developmental time and are supposed to be determined genetically. These parameters determine, for example, how strongly a gene product regulates another, the diffusivity of an extracellular signal, or how strongly a gene product regulates a specific cell behavior according to its concentration.

Cells are represented by nodes in space and these can take a spherical shape, for mesenchymal cells, or a cylindrical shape, for epithelial cells. In addition, the extracellular matrix (ECM) is also represented by spherical nodes. Cell behaviors are represented by discrete rules on these elements (see Fig 4). For example, cell division is implemented as duplication of a cell element.

EmbryoMaker is a modeling framework. By providing a specific gene network and the cell behaviors and mechanical properties it regulates (i.e. a developmental mechanism), EmbryoMaker can simulate arbitrary developmental mechanisms, as it has been done for tooth development [69] and spiral cleavage [70]. To simulate a morphology, EmbryoMaker requires the specification of a developmental mechanism (i.e. a gene network and the regulation of cell behaviors and node properties), and the values of the parameters in such a mechanism and an initial morphology (see Fig 2). All the simulations in this article start from the same very simple initial condition, a small flat epithelial sheet with one gene expressed in a gradient across the sheet. Different developmental mechanisms can have a different number of genes and, thus, a different number of parameters. For example, the two developmental mechanisms depicted in Fig 2, have one parameter for each gene interaction (7 and 10 respectively) and one parameter for the regulation of cell division by gene 6 (in one developmental mechanism) and gene 7 (in the other developmental mechanism).

In the ensemble of random developmental mechanisms, we will also study whether there is a general relationship between morphological complexity and robustness. We will restrict our analysis to robustness understood in the narrow sense of developmental instability. Developmental instability reflects the difference between the morphologies of individuals which share the same genotype and environment [71–73]. Developmental instability can also decrease the efficiency of natural selection because it diminishes the likelihood that adaptive variation in the parents passes to their offspring.

## Results

### Complex morphologies are rare

**Fig 5:**
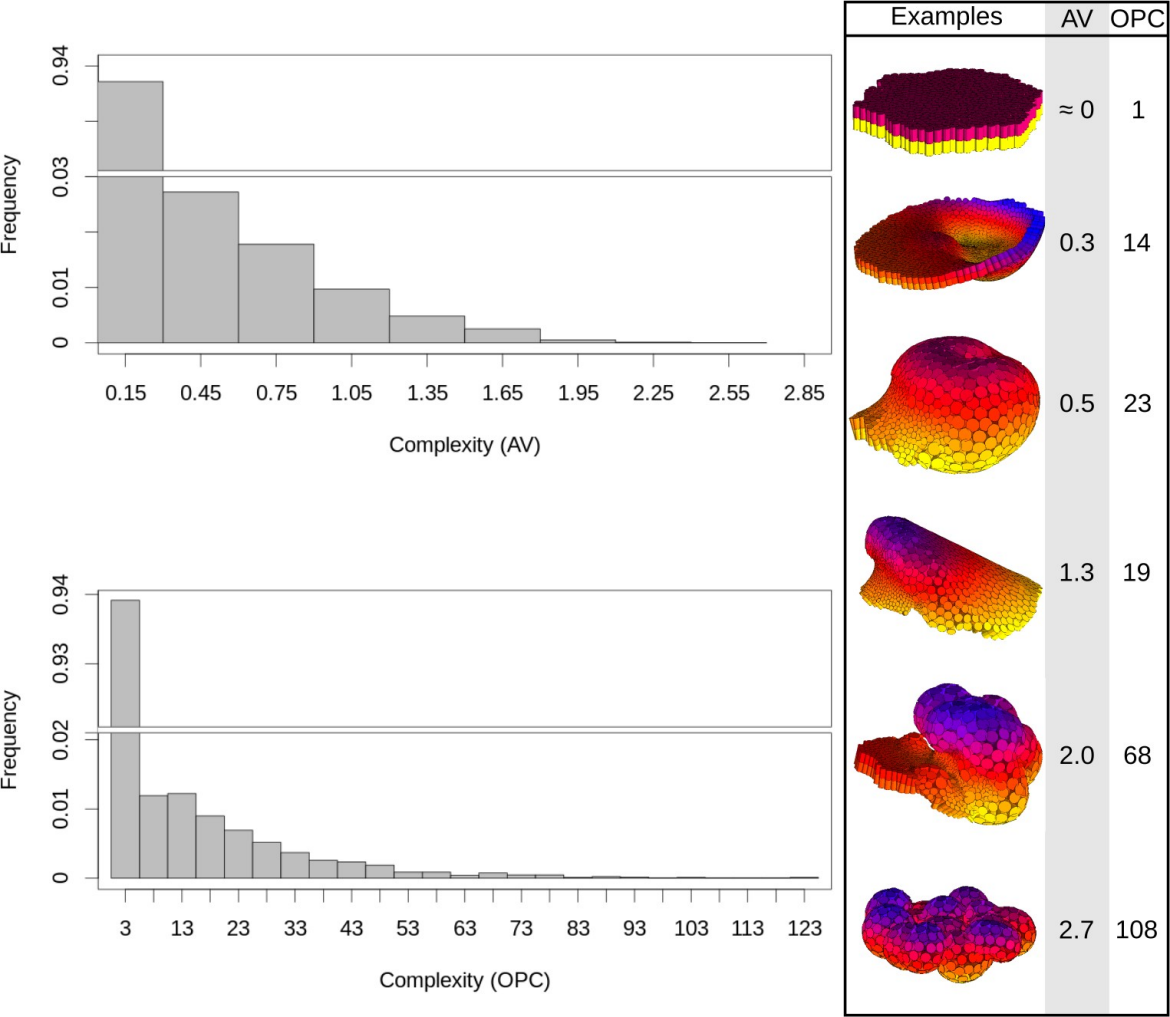
Frequency decreases with morphological complexity. The histograms show the distribution of complexity (for AV and OPC) in the morphologies found in the ensemble. The right panels show example morphologies and their complexities.

We found that some of the developmental mechanisms in the ensemble, i.e. the random developmental mechanisms, were able to produce complex morphologies. The frequency of these morphologies in the ensemble, however, decreases with their complexity (Fig 5). In other words, developmental mechanisms producing complex morphologies are much rarer than the developmental mechanisms producing simple morphologies. In addition, we found that a large proportion of the interactions in each developmental mechanism were not necessary for producing the observed morphology, i.e. the resulting morphology was unaltered if these interactions were deleted. This is perhaps not surprising, given the fact that the developmental mechanisms were randomly built. The superfluous interactions of each developmental mechanism were pruned. The rest of the analysis in this article considers only these pruned developmental mechanisms. Unsurprisingly, the number of non-superfluous interactions necessary for the development of a morphology increases with the complexity of such a morphology (S1B Text).

### Complex morphologies are developmentally unstable

As in a previous study [10], we found that the developmental mechanisms that can lead to complex morphologies tend to have higher developmental instability than the developmental mechanisms that can only lead to simple morphologies (see S1C Text). To measure developmental instability, each developmental mechanism was simulated ten times. All simulations in EmbryoMaker include noise, i.e. random displacements in the position of cells in each iteration, and, then, these ten simulations can give rise to different morphologies. We call each simulation of a developmental mechanism a twin. We took the morphological distance between twins as a measure of developmental instability of the underlying developmental mechanism. The morphological distance between twins was calculated via three methods: 1) as the procrustes distance between homologous cells between morphologies (Homologous morphological distance or HMD); 2) as the average differences in the local convexity between the homologous cells of different morphologies (Convexity morphological distance or CMD); or 3) as the average minimal distances between each cell in a morphology and each cell in the other morphology (Euclidian Morphological distance or EMD; see S1D Text and examples in S1E Text). See Methods 3 for a more detailed description of these measures of morphological distance.

### High morphological complexity requires more finely tuned developmental parameters

**Fig 6:**
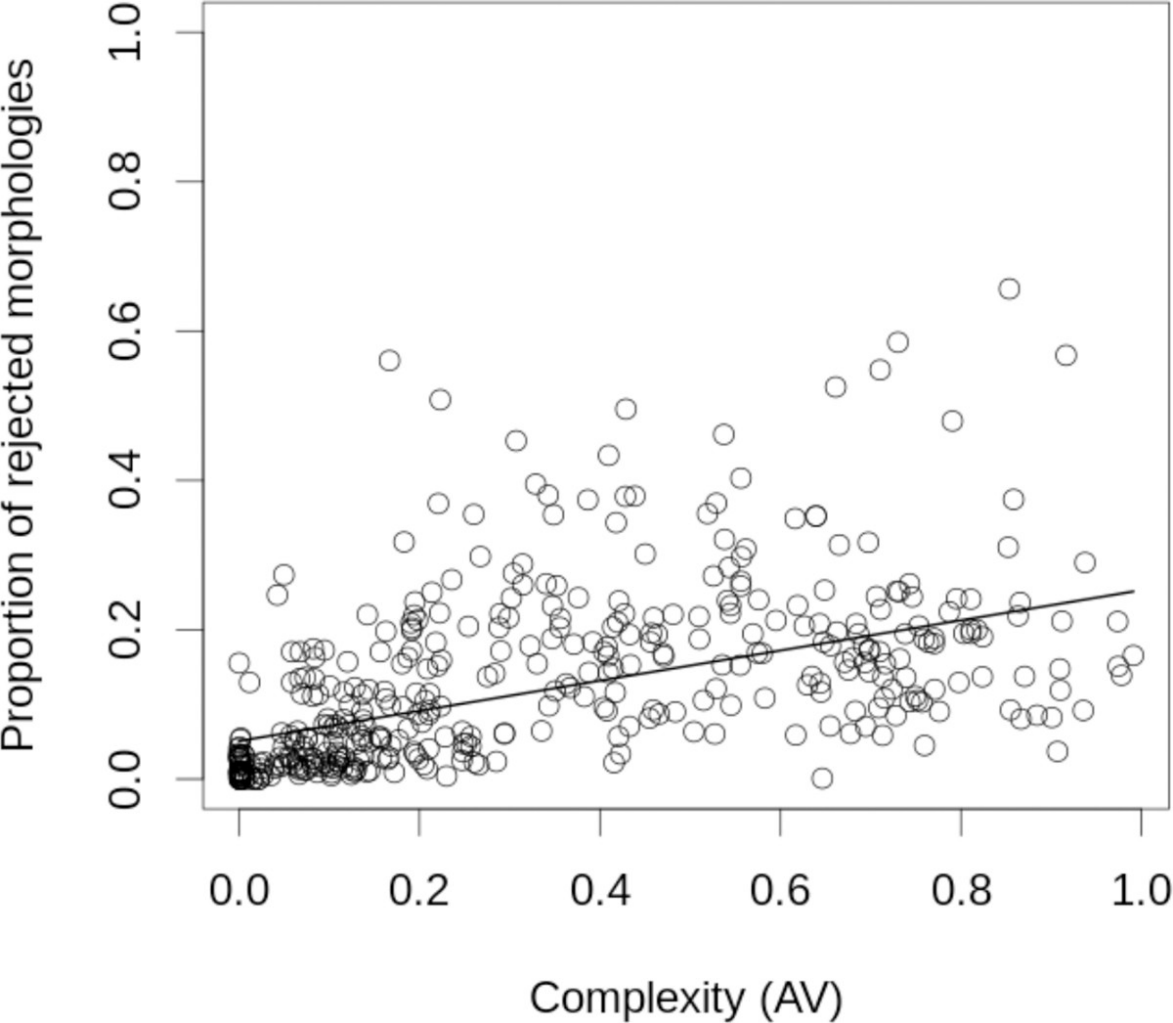
Developmental mechanisms producing complex morphologies occupy smaller regions of the parameter space than developmental mechanisms producing simple morphologies. The figure shows the number of accepted steps in each iso-morphological random walk of each developmental mechanism (Y axis) versus the complexity of the morphology produced by each developmental mechanism. The Y axis, thus, is a measure of the region of the parameter space where a morphology forms. We performed an iso-morphological random walk for the developmental mechanisms in the parental set that are very stable developmentally (i.e. EMD distance between parental twins less than 0.3). In each walk we mutated, one at a time and chosen randomly, gene-gene interactions or gene-cell behavior interactions in each developmental mechanism. If a mutation did not change in a significant way the phenotype (when compared to the original parental morphology in the walk) the mutation was kept, and a new mutation was applied. If the mutation did change the phenotype, this mutation was reversed. This process of mutation was iterated 200 times per developmental mechanism (see Methods 2.3 for details). This way we calculated the proportion of mutations that changed the phenotype: the more mutations changed the parental phenotype, the smaller the region of the parameter space where a developmental mechanism can produce its parental phenotype. We performed 10 random walks per developmental mechanisms. To minimize the effect of random developmental noise in our results each mutant was simulated 5 times (see S1 Text for details). The morphological distance between the final morphology of each of these 5 simulations and the parental was measured using CMD. The average of these distances was used to evaluate whether a mutation was accepted or not. In order to be considered different to the parent, the CMD had to be 0.01 higher than the developmental instability of the parental (measured in CMD). Spearman: pval<0.0001, r_s_ = 0.65, n = 422. See—Methods 2.3. for further details.

Each developmental mechanism in the ensemble was simulated with a specific random combination of parameter values. To better understand these developmental mechanisms, we performed an iso-morphological random walk in some of the developmental mechanisms in the ensemble. We call these developmental mechanisms the parental set. The developmental mechanisms in the parental set were chosen to produce morphologies evenly spaced along the possible range of morphological complexities in the ensemble. In each step of the walk an IS-mutations was introduced to the developmental mechanism. The step was accepted if it did not change the resulting morphology beyond a small threshold morphological distance (see Fig 6 and Methods 2.3). IS-mutations are defined as mutations that change the value of a parameter, but do not change which genes interact, or which cell behaviors are regulated by gene products (i.e. they do not change the number of non-zero parameters [36]). The iso-morphological random walks estimate the region of the parameter space of each developmental for which a specific morphology will form. We found that the simpler the morphology, the larger this region (Fig 6 and S1F Text). Conversely, the more complex a morphology is, the smaller this region. In other words, even when a given developmental mechanism can produce a complex morphology, this is only possible for a small range of values within its developmental parameters. Thus, the most complex morphologies of a developmental mechanism are only possible for small regions of the parameter space of such mechanism. Note that each developmental mechanism may include a different network of interactions and that the strength of each interaction is specified by a different parameter. Based on this the number of developmental parameters can differ between developmental mechanisms (S1G Text).

### The simpler morphology, the larger the number of developmental mechanisms that can produce it

Our study found that there is a global degeneracy in the space of developmental mechanisms, i.e. very similar morphologies can be produced by different developmental mechanisms. This is seen by calculating the morphological distances between morphologies in the ensemble. Degeneracy, however, occurs primarily for simple morphologies and it is very rare for complex morphologies (see S1H and S1I Text). In addition, these figures also show that complex morphologies produced by different developmental mechanisms are more different from each other than the simple morphologies produced by different developmental mechanisms. Moreover, complex morphologies tend to be very different from simple ones. To ensure that these results were not due to the higher developmental instability of complex morphologies, the morphological distances in these figures (S1H and S1I Text) were calculated between the mean morphologies of each pair of developmental mechanisms in the ensemble. This mean morphology was calculated by averaging the morphologies of all the twins of a developmental mechanism in the ensemble (to calculate this average we used the position of homologous cells between the morphologies; see -Methods 3.2). Notice, that simple morphologies can be quite different from each other (see S1J Text), so our results do not stem from how we define complexity.

### The larger the morphological complexity, the larger the mutational asymmetry

**Fig 7:**
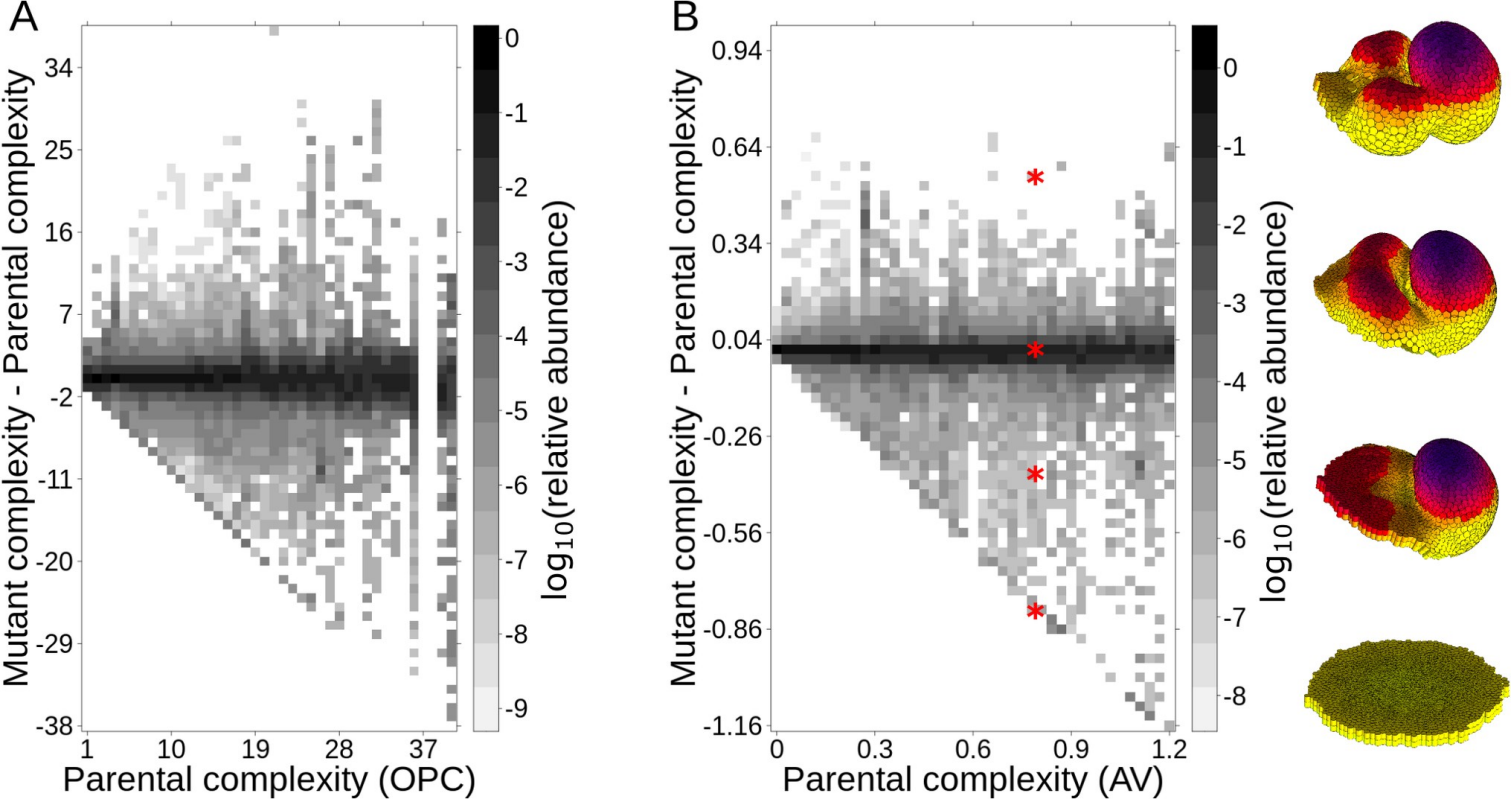
Mutations decreasing complexity are more frequent than mutations increasing it. The plot shows the distribution of the difference in complexity between each parent and its mutant offspring (in the Y-axis) versus the complexity of the parent (X axis). Parents with similar complexity are clumped together in X bins and offspring with similar complexities are clumped together in Y bins. The gray scale in each box indicates which proportion of the offspring (of parents of a given complexity in X) exhibit a given complexity. The plot shows that most offspring have a complexity similar to that of their parents. It also shows that, for complex morphologies, there are more offspring that are simpler than their parents than offspring that are more complex than their parents (i.e. there is more gray for lower Y when X is large). Notice that even the most complex parents can have very simple offspring but that simple parents rarely have very complex offspring (see also S1L–S1N Text).

To study mutational asymmetry, we performed a mutational screening of a random sample of developmental mechanisms in the ensemble (Methods 2.2). Each developmental mechanism in the ensemble, what we call a parent, was IS-mutated to produce offspring developmental mechanisms. EmbryoMaker was then used to obtain the morphology of each offspring (Fig 7 and S1K Text, S1 Text). Each mutant differed from its parent in only one parameter value. Fig 7 shows the offspring complexities versus the parent complexities (see also S1L–S1N Text). The figures show a clear mutational asymmetry. The higher the complexity of the parent, the larger the proportion of offspring that are simpler than their parents. Most of the developmental mechanisms producing complex morphologies in the ensemble had some offspring with the minimal possible complexity (a flat epithelium). Thus, many complex morphologies are one mutation away from the simplest morphologies. However, the reverse is not true as the vast majority of simple parents were not one mutation away from producing complex morphologies (see Fig 7 and S1K–S1N Text). This mutational asymmetry was even more evident when the mutational analysis was done with Topological or T-mutations (see S1L Text). T-mutations are defined, as in [36], as mutations that change which genes interact with which other genes, cell behaviors, or cell mechanical properties, i.e. the network of the developmental mechanism is modified.

Offspring was generated by mutating each parameter of the parent. Each parameter was IS-mutated the same number of times and each mutant offspring had only one mutation. The magnitude of these mutations was proportional to the value of that parameter in the parent and ranged from -80% to +80% in 20% intervals. (A) uses OPC complexity and (B) AV complexity. Offspring is arranged along the y-axis according to their complexity minus their parental complexity and along the x-axis according to the complexity of their parents. The x-y plane is divided in square bins. For (A) the size of each bin is 0.03 AV units, for (B) of 1 OPC unit. The darkness of each bin represents the natural logarithm of the relative abundance of offspring of a given parental complexity (x-axis). To calculate the relative abundance, for each column (x-axis), we divide the number of offspring falling in each bin by the total number of offspring in that column. Thus, the relative abundance of each column in a plot sum 1. On the right we show some examples of offspring morphologies. The red asterisks mark the complexity of the examples. See S1 Text for details.

### The more complex the morphology, the more complex the GPM

**Fig 8:**
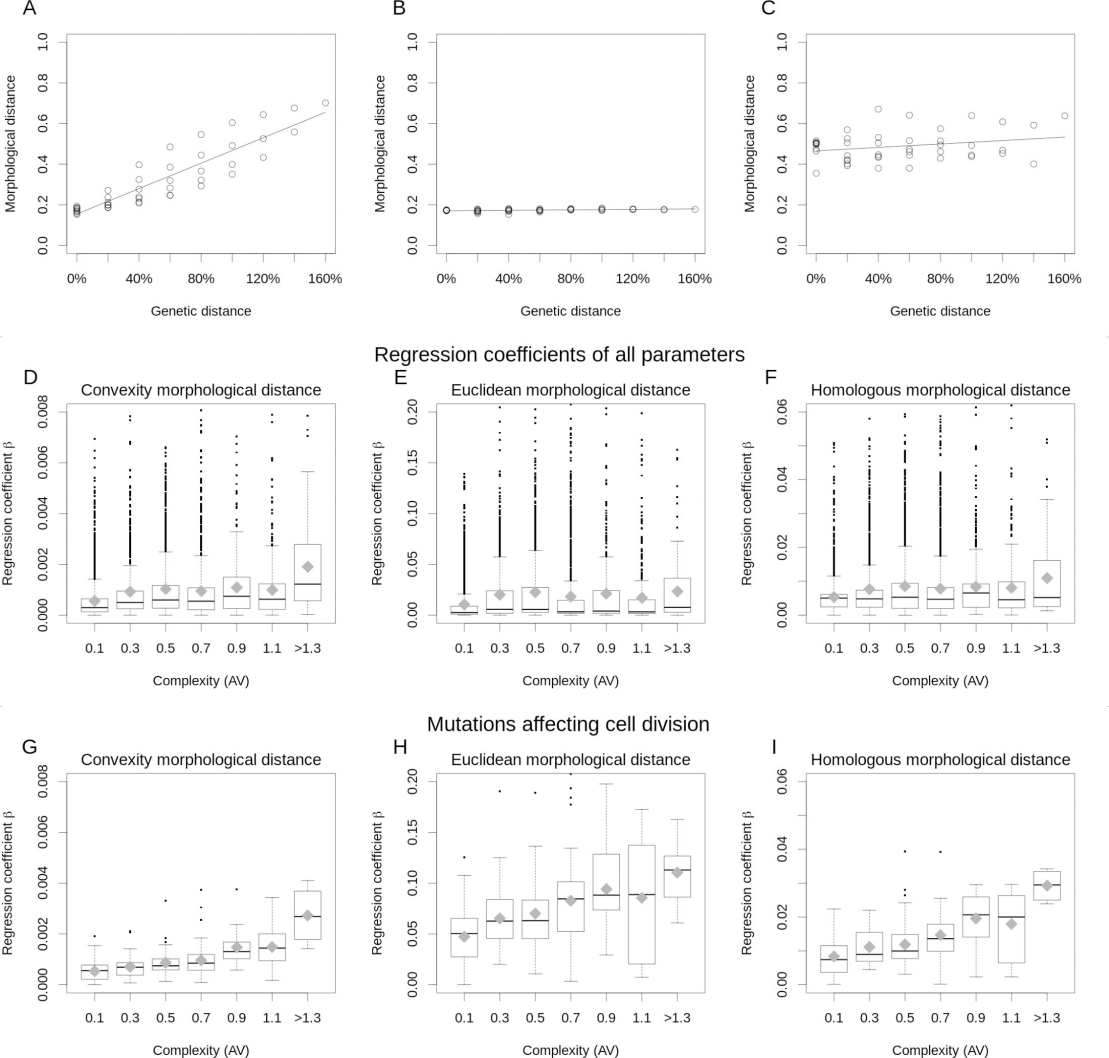
Complex morphologies have more complex GPMs. (A-C) example of “GPM regression plots”. The X-axis shows the “genetic distance” between each pair of mutant offspring of a given parent. Each offspring diverges from its parent in only one parameter and in different proportions of the parent parameter value (from -80% to +80% in 20% intervals). The “genetic distance” is the change in a parameter value (in proportion to the value in the parent) between each pair of mutant offspring. For example, in the 0% position in the x-axis we have all the twins of each mutant offspring. The 0% axis position, thus, indicates developmental instability. In the 20% position we compare the mutants that are at a 20% genetic distance from each other. This is, for example, the mutants that are at a 40% distance to the parent versus the ones that are at a 20% distance to the parent, the ones that are at 40% versus those that are at 60%, etc. Each point in the Y axis shows the average morphological distance between all the offspring with a parameter value and all the offspring with another parameter value at a specific genetic distance. The morphologies in (C) have a higher developmental instability than the morphologies on (B), but since the morphological distances between offspring do not change with genetic distance, they both have very small regression coefficients. (D-F) Plots of the GPM regressions coefficients of each parent and parameter against the parent complexity. In other words, the slopes of the GPM plots (as the ones shown in A, B and C) are plotted against the complexity of the parent. In this plot there would be one point per parent and parameter but those are binned into boxes of 0.2 AV complexity intervals. Boxes enclose 50% of the regressions per parent interval (i.e. for all the parameters of all the parents in an interval). The line in the box shows the median and the gray diamond the average for that interval. The whiskers extent 1.5 times the interquartile range of the box. (G-I) As in (D-F) but considering only the mutants that directly affect the cell phase cell property, *P*^*PHA*^, and, thus, affect cell division rates. Spearman correlations. (D) r_s_ = 0.2635, pval<0.001, n = 7009. (E) r_s_ = 0.1585, pval<0.001, n = 7009. (F) r_s_ = 0.0781, pval<0.001, n = 7009. (G) r_s_ = 0.5307, pval<0.001, n = 691. (H) r_s_ = 0.4315, pval<0.001, n = 691. (G) r_s_ = 0.5429, pval<0.001, n = 691. See section 6 in S1 Text.

To study the GPM we use the mutational screening of the previous section to calculate the regression between genetic and morphological distances among the IS-mutant offspring of each parent developmental mechanism (as in Fig 7). See Fig 8A–8C for examples of such GPM regressions. We calculated one GPM regression per developmental mechanism and parameter (i.e. for a parent and all its mutant offspring in one parameter). The distance in parameter values between two mutants of the same parent was taken as a proxy for their genetic distance (details in Fig 8 and S1 Text). We call a GPM “complex,” if small genetic changes often lead to large morphological changes and, therefore, morphological distance increases rapidly with genetic distance. In that case, the GPM regression coefficient is relatively large. Conversely, the GPM regression coefficient should be small if morphological distance increases slowly with genetic distance (a simpler GPM), most mutations have small gradual morphological effects. In addition, such a regression should also be small if developmental instability is so large that twins are as different from each other as they are different to their non-twin brothers or parents. In this latter case, as seen in Fig 8C, the low regression arises from the noisiness of the plot. In addition, such a regression should also be small if developmental instability is so large that twins are as different from each other as to the other non-twin mutant offspring or parents. In this latter case, as seen in Fig 8C, the low regression arises from the noisiness of the plot.

In principle, a low GPM regression coefficient in a parameter could also arise from a highly non-linear GPM. However, non-linear regression measures are unlikely to be robust to noise and the small number of points per parameter in our study. It should also be noted, the form of these non-linearities could be different for each developmental mechanism and parameters. Thus, even if enough points would be available, it would be difficult to compare the different parameters and developmental mechanisms. This is not the case with a linear regression. Note also that the y-axis in the GPM plots is the morphological distance, not the value of any morphological trait, and that two morphologies can be quite different from each other, yet at the same morphological distance from a third (e.g. the parent).

Fig 8D–8I shows the GPM regressions coefficients for each parameter of each parent versus the complexity of the parent (see also S1O–S1R Text). Each offspring was simulated 10 times to control for noise. Fig 8 shows that the complexity of the GPM increases with the complexity of the morphology. The higher the complexity, the larger the morphological changes produced by small genetic changes.

The result in Fig 8 is not due to the higher developmental instability of complex morphologies. If that were the case, the morphological distance between twins would be roughly as large as the morphological distance between brothers (i.e. offspring from a same parent but with different values in their parameters). For the developmental mechanisms producing complex morphologies, the GPM regression coefficients would necessarily be small, as described above. That is the contrary of what we observe and, thus, the developmental mechanisms producing complex morphologies have inherently more complex GPMs.

We found that many developmental mechanisms had parameters that could be changed without leading to any morphological change (see Fig 8B), even after totally superfluous interactions were eliminated (see first section of the results). We also found that some of the interactions associated with those parameters could not be deleted without the morphology changing dramatically. In other words, these interactions are not superfluous, but rather required for the development of a morphology. However, they do not contribute much to the morphological variation. Based on this, we re-analyzed the GPM plots for the parameters of each developmental mechanism that have a large contribution to morphological variation, e.g. the parameters with larger regressions in each developmental mechanism. These show an even clearer relationship between morphological complexity and the regression coefficients of the GPM (S1M and S1N Text). The same occurs if we only focus on the GPM regression for the proliferation rate parameter (i.e. how strongly cell division is affected by at least one gene in a developmental mechanism), which tends to have a strong effect on complexity (Fig 8G–8I and S1O Text).

### More complex morphologies lead to a larger diversity of morphologies when mutated

The result that the GPM plots of complex morphologies have larger regression coefficients implies that more diverse morphologies are accessible by mutation from complex morphologies. In other words, if the regression is high it means that mutants are more morphological different from each other, thus overall there is a higher diversity of morphologies. This means that a larger disparity of morphologies is possible from genetic variation in the developmental mechanisms that can lead to complex morphologies than in the developmental mechanisms that cannot.

## Discussion

Our results can be summarized as follows: 1) Most developmental mechanisms do not produce complex morphologies; 2) Those that do, can only produce them for a relatively narrow range of their parameter values; 3) complex morphologies are developmentally unstable; 4) Simple morphologies can be produced by different developmental mechanisms while complex morphologies often cannot; 5) Complex morphologies are more different from each other than simple morphologies; 6) Mutational asymmetry is common and increases with morphological complexity, that is mutations are more likely to decrease than increase complexity; 7) Developmental mechanisms that lead to complex morphologies tend to have more complex GPMs than developmental mechanisms that lead to simple morphologies; and 8) The developmental mechanisms of complex morphologies, when mutation occurs, can lead to more diverse morphological variation than the developmental mechanisms that can only produce simple morphologies.

Some of our results were also found in GPM models at other phenotypic levels. This is an interesting result since different phenotypic levels are quite diverse and form via very different mechanisms (e.g. gene and cell interactions in development versus hydrogen-bond nucleotide interactions for RNA secondary structures). Moreover, our model differs from other models, like RNA models, because it does not include a proper genotype, but a set of parameters, as utilized in gene network and other development models [51–62]. In this discussion we will call the specific combination of parameters in an individual a genotype (i.e. a developmental mechanism with specific values in each of its parameters). An additional difference between our model and most other GPM models is that the dimensionality of the genotype and the phenotype is not fixed. In other words, the ensemble has developmental mechanisms with different number of genes and interactions and morphologies with different number of cells (each of them can vary along the x, y, and z coordinates and, thus, there are *3N*_*c*_ dimension per morphology, where *N*_*c*_ is the number of cells).

One property found in most, if not all, GPM models is that different phenotypes have very different frequencies [41–50,74–76]. That is, some phenotypes are associated with many different genotypes while others are associated with only a few genotypes. This is also the case in our study; however, in our study we also found that the common phenotypes happen to be simple and the complex phenotypes happen to be rare. Three other studies have found a similar relationship between frequency and phenotypic complexity [74,76,77]. These studies also found that the individuals with complex phenotypes tend to mutate into a larger diversity of other phenotypes than the individuals with simple morphologies. The earliest of these studies uses a computational model of the development of a specific organ (teeth) [74]. The second study is a model of small mutable computer programs [76]. The first study, thus, applies only to a specific organ and the related developmental mechanisms, while the second model has no direct biological analogue. The third study [77] provides some analytical arguments for the lower frequency of complex morphologies and shows that this applies to four relatively simple GPM models; the RNA model, a finances model, a model of circadian clocks, and a very simple model of branching in plants.

Another property found in many GPM models [41–45,47–50,75,76] is that the neutral networks of the most common phenotypes are intertwined and percolate the genotypic space. Neutral networks are sets of genotypes that lead to the same phenotype and that can be transformed into each other through simple mutations that do not change that phenotype [40–43,47–49,75,76,78]. The intertwining and percolation of the common phenotypes means that genotypes of rare phenotypes can be transformed into the genotypes of common phenotypes by just one, or few, mutations [39–42,44,49,79].

**Fig 9:**
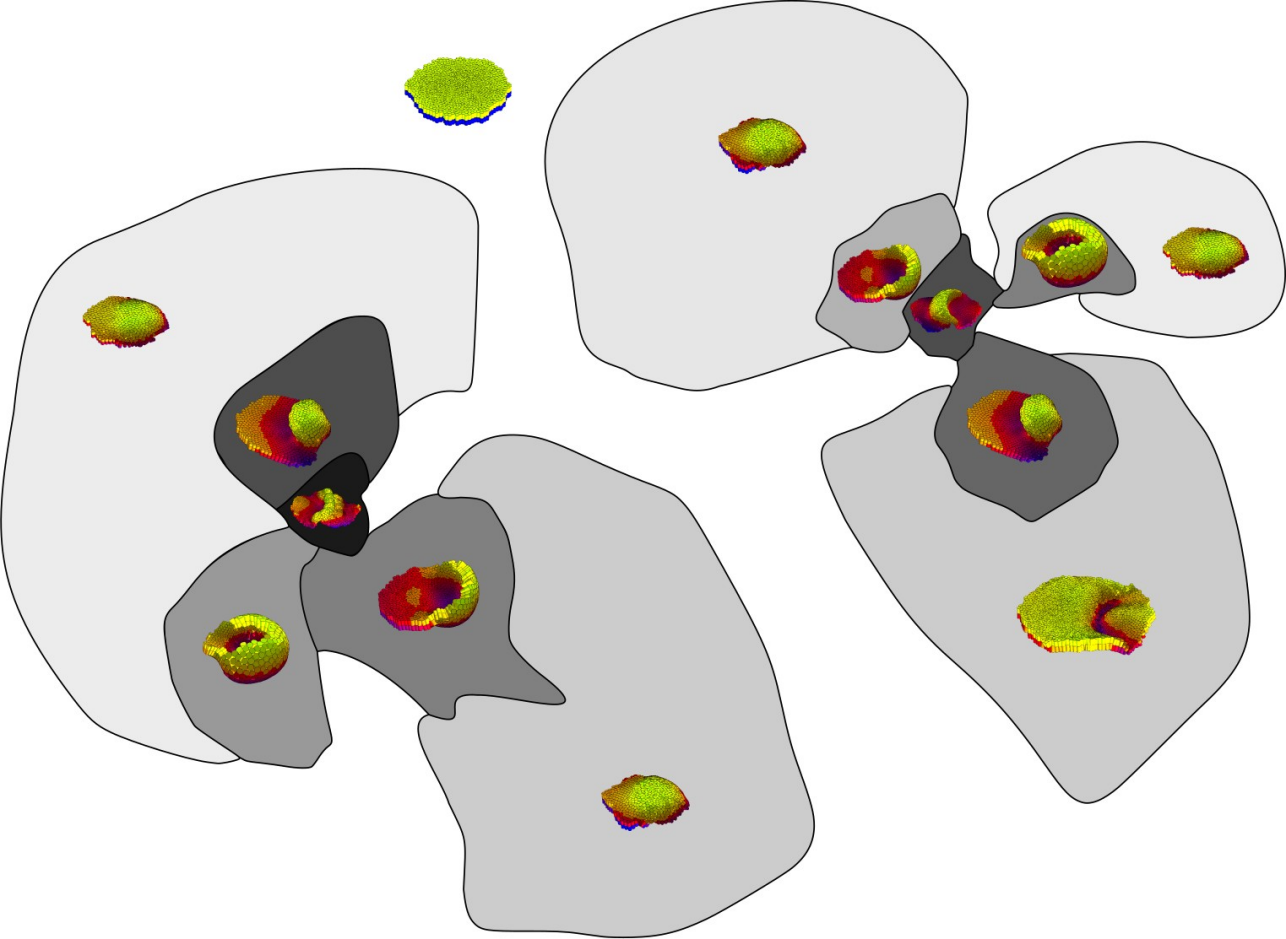
Idealized schema of the developmental parameter space of a developmental mechanism and the regions occupied by simple and complex morphologies. Idealization of the developmental parameter space of developmental mechanisms. Each colored region represents a parameter region (i.e. neutral network) where a morphology of a given complexity would form. As in our results, the simpler morphologies occupy larger regions of the space and most such regions are in contact with the region producing the simplest morphology (in white). The regions with complex morphologies tend to neighbor regions that also produce complex morphologies.

In our development model, there is also intertwining and percolation of the neutral networks of the most common phenotypes, that is the simplest ones. In fact, the simpler the morphology the larger its neutral network (as shown in the iso-morphological random walks, Fig 6). In our case, any genotype is only one or few mutations away from a genotype of a simple morphology. In other words, most morphologies can be transformed into very simple morphologies by a single mutation in the underlying developmental mechanism. However, there is a strong mutational asymmetry. Most simple morphologies cannot be transformed into complex morphologies by a single mutation in the underlying developmental mechanism. In fact, in most cases, many mutations would be required for that to occur, i.e. change to a different gene network topology and developmental parameters values would be required. This is the mutational asymmetry shown in Fig 7. In addition, we found that complex morphologies tend to form clusters in the parameter space (i.e. the neutral network of each complex morphology is close to the neutral network of other complex morphologies). This is because complex morphologies can be transformed into very simple ones by a single mutation, but they can also be transformed into slightly less complex morphologies. The latter, however, are not as likely to be transformed into the former by single mutations (due to the mutational asymmetry). These slightly less complex morphologies are found in large areas of the parameter space and are made possible by many more developmental mechanisms. Thus, the parameter space can be seen as having a structure in respect to morphological complexity (see Fig 9). Complex morphologies form clusters of neutral networks, with the less complex morphologies bordering the more complex, while all of them are in contact with the neutral networks of the simplest morphologies.

To our knowledge no previous study has explored the relationship between morphological complexity and developmental instability, or between morphological complexity and the complexity of the GPM.

### Evolutionary implications

One evolutionary implication of our results is that in those lineages where complexity increases in evolution, it does so at a progressively slower rate as complexity increases. Thus, on one hand, as complexity increases it becomes more difficult to change development to produce even more complex morphologies (due to the mutational asymmetry), and on the other hand, this complexity is less likely to be passed between generations and selected (due to a complex GPM and higher developmental instability). It follows that the evolution of morphological complexity would gradually slow until it effectively stops.

Our results also imply that the evolution of complex and simple morphologies is qualitatively different. Complex morphologies evolve under a complex GPM and higher developmental instability. This reduces the efficiency of natural selection. From a classic neo-Darwinian paradigm this would imply that complex morphologies should evolve less or more slowly, at least [20,38]. Complex morphologies produce a higher morphological diversity, e.g. higher disparity [31], than simple morphologies for the same amount of genetic variation. In other words, the offspring of complex individuals spread across large regions of the morphospace. This higher morphological disparity allows, in principle, for adaptation to a larger diversity of selective pressures on morphology (as suggested previously in studies based on tooth development [80)]. Thus, the differences between complex and simple morphologies cannot be reduced to differences in evolution rates. The differences are in the tempo and mode of evolution. Simpler lineages can evolve faster, but only within a smaller region of the morphospace, while complex lineages may evolve in wider regions of the morphospace. The observed evolutionary rates would then depend on the coarseness of the selective pressures on morphology (e.g. selection for precise small changes in a single trait versus selection for the general features of overall morphology), the time scale considered, and how changes in morphology are actually conceptualized and measured.

It is relevant to stress that the evolutionary differences we encounter between complex and simple phenotypes are not due to natural selection or to any force that could be identified from a classical neo-Darwinian or modern synthesis approach to evolution. Our results are a mathematical necessity or constraint that can only become apparent once one considers how gene and cell interactions can be organized into networks to lead to pattern formation. In other words, the evolutionary differences between complex and simple morphologies are inherent to development given the range of cell behaviors and the physical and logical properties of animal cells [81]. These properties may themselves evolve over very long timescales but, on the timescale of animal evolution, they should be considered inherent to development.

One main conclusion of our study is that the evolution of morphological complexity becomes progressively slower as complexity increases. This does not imply that morphological evolution would eventually stop. Morphological complexity is unlikely to be selected *per se*, therefore morphology will keep evolving, it may simply not evolve to become more complex. In addition, an effective limitation to morphological complexity may not preclude the evolution of complexity in other aspects of the phenotype [82]. For example, metabolism, behavior, and culture have their own mechanisms with their own types of interactions [83], which determine their possible phenotypic variation. These other phenotypic levels may be able to evolve in complexity even in those lineages where morphological complexity has reached a limit. At these other levels the phenotype is also constructed from genetic, epigenetic, and environmental information in a process analogous to development (e.g. cognitive development and learning). It is then possible that limitations analogous to the one we study, also apply to these other phenotypic levels. Then, at each phenotypic level, complexity may become more difficult to evolve as it increases.

### Caveats

First, it can be disputed that randomly folded epithelia are not really equivalent to complex animals. Accordingly, we do not find many animal-like morphologies among the morphologies we consider complex. The way we measure complexity, seemingly random morphologies are sometimes classified as being more complex than morphologies that might look more animal-like at first sight. However, in order to avoid this, we would require a complexity measure that can distinguish between biological and not-biological complexity, a difficult task in and of itself. In any case, both random and animal like morphologies result in high complexity values using our complexity measures.

Second, we only simulated embryos with up to 5000 cells and maximal 10 genes, since otherwise the computational simulations would take too long. To shorten computational time, we also modelled each cell as a single cylinder or sphere. This precludes us from simulating planar polarized cell contraction, since simulating that contraction would require that different parts of the cell contract at different rates and thus, requires that cells are made of several cylinders or spheres. The same applies to planar polarized cell adhesion. The complexity of the morphologies we find may be limited by this and by the sizes of the embryos we simulate.

Third, for simplicity, our model considers the ECM as made of spherical nodes. This precludes an accurate simulation of the basal lamina. However, we acknowledge the importance of the basal lamina in epithelial morphogenesis. One of the most important effects of the basal lamina on the physical properties of epithelial tissues is the stiffness it provides [84]. The stiffness comes from a planar network of proteins, mainly collagen IV, which is stabilized by sulfilimine chemical bonds [85]. Instead of modeling the basal lamina as ECM with this property, EmbryoMaker approaches the stiffness of the basal lamina through the way it models epithelial cells. Epithelial cells are modeled as cylinders, with a basal and an apical side, which have a tendency to avoid being bent. By regulating this tendency, epithelia with different stiffnesses are simulated in the ensemble (see *p*^*ERP*^ and *p*^*EST*^ variables in the model description). This is just an approximation and it is quite likely that a more accurate implementation of the basal lamina would increase the repertoire of morphologies obtained in our simulations.

Fourth, a similar caveat applies to the mesenchyme. Although we simulate the mesenchyme, we do not consider its morphology in our complexity analysis because, in all embryos in the ensemble, such a morphology mimics that of the epithelium or it is very noisy. This implies that our approach is not considering the morphologies that can arise from morphogenesis in mesenchymal tissues. This limitation also applies to the non-epithelial tissues (e.g. the blastomers in the blastula of many species) that EmbryoMaker simulates as being made of spheres as the mesenchyme (e.g. as in our article on spiralian cleavage [70]). Thus, for example, 3D condensates, rods and lumens within solid 3D tissues are not found in our ensemble.

Fifth, the morphologies we found in the ensemble are simpler than those of most animals. It is likely that to attain the complexities compatible with those of metazoa we will need a different approach. A natural option would be to simulate morphological evolution and development in a unified model, as done for simpler models [58,86]. This is likely our next research aim. However, this aim is even more computationally demanding, since it requires simulating large numbers of genetically similar developmental mechanisms for a large number of generations. The individuals in a population only differ in a small number of parameter values. Thus, evolutionary simulations explore a smaller area of the space of possible developmental mechanisms than ensemble simulations for the same amount of computation. In addition, evolutionary simulations depend on what is being selected and proposing what has been selected in the morphology in the past is not a trivial task.

Sixth, natural developmental mechanisms are the result of a historical process of evolution, and, thus, not random. However, studying random networks is a methodological choice. Although our ultimate goal is to explain the developmental mechanisms existing in nature, having some understanding of the possible ones may help to discern which properties of existing developmental mechanisms are logical necessities (i.e. logical rules on how genes need to be wired for morphogenesis to occur) and which are due to historical accidents and dependencies.

Seventh, the developmental mechanisms we found in the ensemble may differ in many ways from real ones. One important difference may stem from the fact that we are only looking for developmental mechanisms that are able to produce complex, robust morphologies. In reality, each gene network, or part of its genes, can be involved in several functions at the same time (e.g. engaged in morphogenesis, but also in ensuring that specific cell types differentiate in a specific location within the morphology) [68]. Thus, we may be encountering developmental mechanisms that are much simpler than the ones in real animal development. In fact, relatively complex developmental mechanisms are found even in the early development of relatively simple metazoa [87]. Moreover, due to our limited sampling, we are only finding the simplest developmental mechanisms that can lead to any given morphology in the ensemble. On account of developmental systems drift [88] these may not always be the ones found in animal development. Most importantly, current developmental mechanisms have evolved from previous ones and there may be biases or rules on how these can evolve (in addition to mutational asymmetry). For example, some metabolic and protein interaction networks have been statistically analyzed to show that their statistical properties can be explained by preferential attachment rules (e.g. genes with many interactions are more likely to get further interactions when new genes are introduced in the network by mutation) [89,90]. These studies suggest that these statistical properties enhance the robustness of networks to mutation [89,91]. For most organs, the networks involved in morphogenesis are not very well characterized [68]. This makes it unclear if they also show these properties. In addition, some previous studies [57–62] show that the capacity to develop complex morphologies, or even just complex patterns of gene expression in space, depend on the specific topology of each network rather than on its bulk statistical properties. Similarly, robustness to mutation may not necessarily relate to the developmental instability we study with our model.

Other authors suggest different “rules” for the evolution of developmental mechanisms. Some suggest that there is a limited number of ways in which gene products, cells, and cell biophysical properties can be wired into developmental mechanisms for the development of complex morphologies [57,58,80]. Then, the evolution of development may be understood as the replacement between these developmental mechanisms in each body part of each species [58,80]. Other authors relate the evolution of complex morphologies to the progressive recruitment, in some animal lineages, of different cell behaviors and cell biophysical properties [67,92].

Many of the caveats above imply that there are some realistic morphologies that are not found in our ensemble. In principle there is no reason to expect that the morphologies that were not found have properties that are very different from the properties of the morphologies that were found in the ensemble. These morphologies may show a similar relationship between complexity and frequency, between complexity and developmental stability, and between complexity and mutational asymmetry, etc. In the next section we argue why this is likely to be the case and why our results may be general for animal development and perhaps even beyond.

### Why is complexity rare and why is there a mutational asymmetry

**Fig 10:**
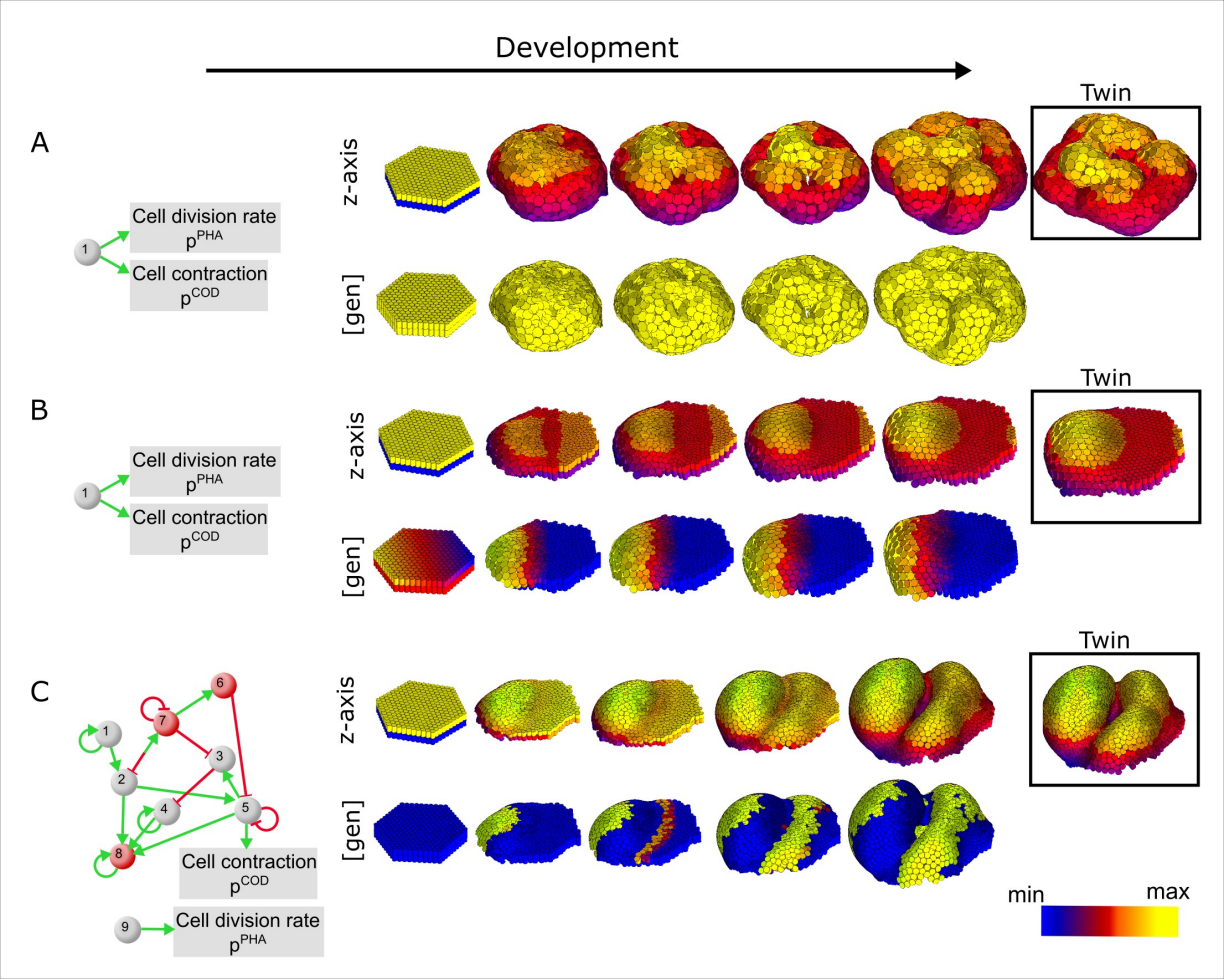
Simple examples of developmental mechanisms. The left column shows the developmental mechanisms that lead to the changes of the initial morphology (second column) into a final morphology (last column). The upper row in (A-C) show the z-axis position of each cell in a color scale. The lower row shows in color the concentration of a gene that regulates cell contraction. The box in the right shows the morphology of a twin of the morphology just left to it: these are two morphologies arising from the same developmental mechanism. The differences between them are due to noise in development. (A) A homogeneously expressed gene regulating cell contraction can lead to a complex, yet unstable morphology (see twin in the box). (B) If the gene regulating cell contraction is expressed in a gradient, a single evagination of increasing curvature will form. (C) Example of a developmental mechanism that leads to a spatial non-monotonic change in the expression of a gene that regulates cell contraction. This leads to a relatively complex morphology that is stable because it is partitioned into several, relatively small, territories of gene expression.

Development starts from morphologies that are simple, e.g. a zygote cell. In our simulations, development starts from a flat epithelium. For the initial morphology to become more complex, cells have to change their relative position. In other words, they have to move. Cell movement requires the regulation of cell behaviors, e.g. cell contraction. In addition, the cells in different parts of a morphology have to move in different directions so that the position of each cell is not easily predictable from that of its neighbors. This difficulty to predict cell positions is precisely what makes the morphology complex. A recent study using a very similar ensemble approach [10] suggests that development towards complex morphologies can be achieved by two alternative options. Either by activating the same cell behaviors in all the cells of a morphology or by activating cell behaviors differently in different parts of a morphology. In the first option, even if all cells activate the same behaviors in the same way, different cells end up moving in different directions and complex morphologies develop (see Fig 10 and [10]). The morphologies produced in this way are very sensitive to noise (see Fig 10A) and, thus, we only consider the option wherein cells in different parts of a morphology behave differently.

**Fig 11:**
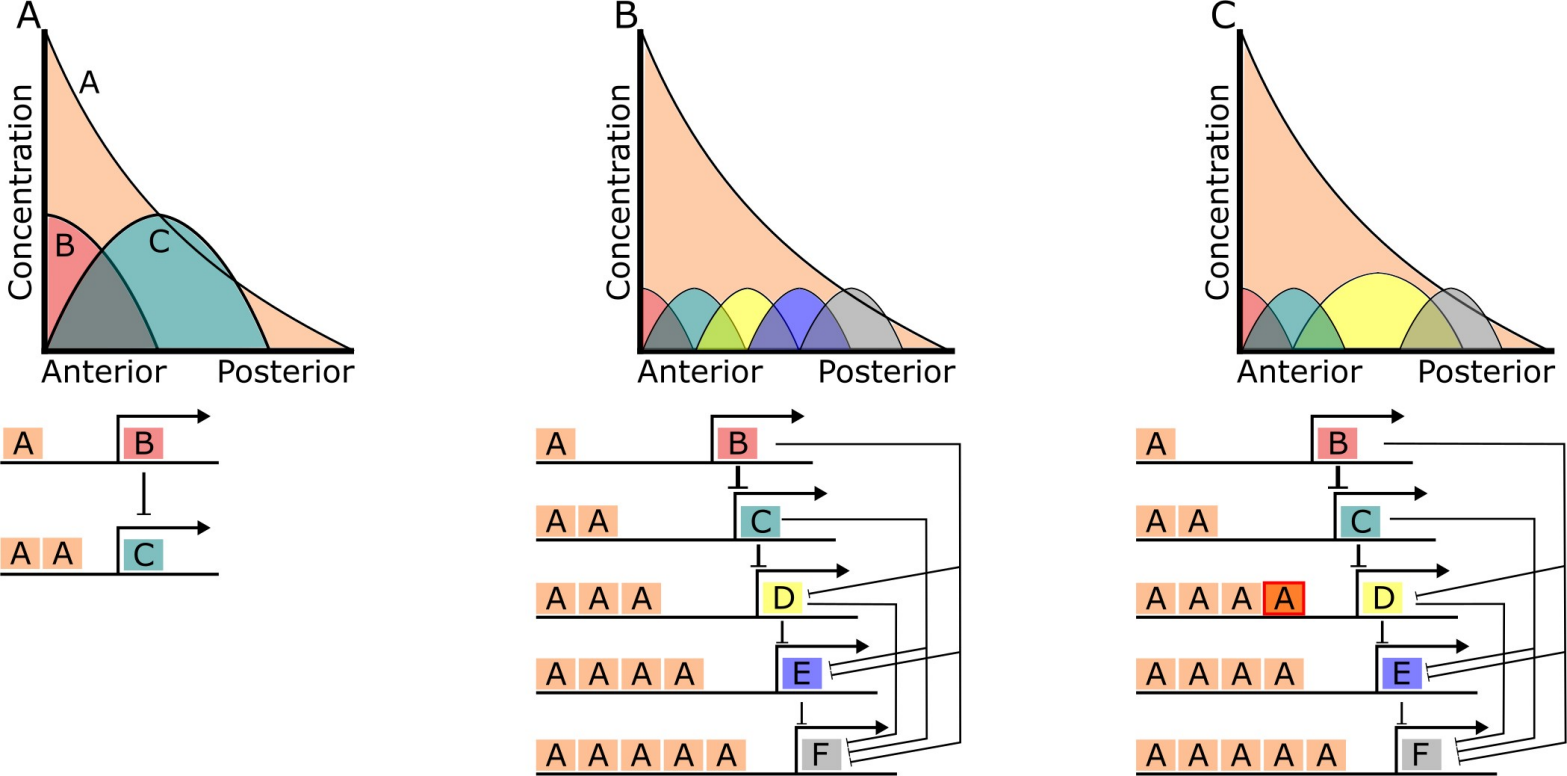
Example developmental mechanisms. (A) The plot shows the concentration of different gene products at different distances from the source of A. For simplicity we do not consider the signal transduction pathway of A but we consider that this pathway leads to a dose-dependent increase in the concentration of a transcriptional factor that binds to a specific enhancer “A”. The network below represents a schema of the network. The A boxes represent enhancers for the transcriptional factor induced by A. B inhibits C’s expression. The genes with a higher number of A enhancers would be able to be expressed at lower concentrations of A. (B) as (A) but for a more complex network leading to several distinct territories of expression. (C) as (B) but D has acquired a larger affinity for the transcriptional factor activated by A and then, it totally inhibits the expression of E. A similar developmental mechanism seems to be acting in the early dorso-ventral patterning of *Drosophila* [94].

Typically, for cells in a morphology to behave differently, various genes have to become expressed in such cells (or the same gene must be expressed at different levels at different parts of the morphology). In other words, different spatial territories of gene expression must form in a morphology. This requires that cells communicate to change each other’s gene expression, e.g. through the secretion and reception of extracellular signals. The diffusion of an extracellular signal generates spatial asymmetries. Cells at different distances from the cells secreting the signal (i.e. the source of the signal) experience different concentrations of such signal (see Fig 11). These asymmetries can then be used to generate new spatial territories (i.e. the expression of genes in new spatial patterns). This can happen in various ways. For example, differences in the concentration of a single signal over neighboring cells can be used to express various genes over a range of distances from the signal’s source [93]. However, each such a gene would require a specific sub-network of gene interactions for this to occur. Imagine that each cell can transduce the extracellular signal into the expression of a transcriptional factor in a dose-dependent manner (see Fig 11). If the promoters of various genes have different affinities for that factor and can only be expressed if the factors bind with some threshold frequency, these genes would be expressed at different distances from the signal sources (e.g. the genes with low affinity promoters would only be expressed in cells close to the signal’s source where the concentration is high). In addition, if each gene represses the genes that have higher affinity promoters, then each gene becomes expressed in a territory at a specific distance from the signal’s source (see Fig 11).

In the previous example, it is clear that each territory requires specific patterns of promoter affinity and specific patterns of inhibitory interactions with other genes. In addition, the parameters of such interactions need to be within a specific range, otherwise some of the territories would not form (e.g. the promotor affinities and inhibitory interactions need to be strong enough; see Fig 11C). These requirements are not specific to this developmental mechanism, rather they are general. In reaction-diffusion Turing-like mechanisms, for example, the formation of many regularly arranged territories can occur if there is specific network of interactions between genes and these are within a specific range of values [95,96]. However, the territories formed by those mechanisms are very similar to each other (i.e. the same genes expressed at the same levels). Thus, the formation of different types of territories requires the recruitment of additional gene interactions or whole additional developmental mechanisms (e.g. the combination of two reaction-diffusion mechanisms or the combination of reaction-diffusion mechanism with mechanisms as the ones depicted in Fig 11; [58]).

The development of complex morphologies requires the formation of different territories, but it also requires that the cell behaviors and cell mechanical properties in each territory are within a specific range of values (e.g. too much of a cell behavior like cell division may break the epithelium). Notice, that in the development of complex morphologies different parts of the morphology may mechanically interact and send extracellular signals to each other, and that the acceptable range of values in a parameter depends on the values in many other parameters [64].

Our result that complex morphologies are rare in the ensemble can easily be understood from the above argument. The more complex is a morphology, the more it requires developmental mechanisms with many interactions arranged in very specific ways and with very specific parameter value ranges. Most randomly constructed developmental mechanisms do not fulfill these requirements and, thus, most developmental mechanisms do not lead to complex morphologies, which explains the low frequency of complex morphologies.

A similar argument can be used to explain the mutational asymmetry. Since the developmental mechanisms that lead to complex morphologies require many interactions to be arranged in specific topologies and specific ranges of parameter values, it follows that there are more targets for mutation to disrupt these complex requirements and consequently lead to a simplification of morphology. On the contrary, an increase in complexity requires adding interactions in very specific ways and with very specific parameter values and, most mutations are unlikely to lead to an increase in complexity. We think this is a very general asymmetry that could be found in many processes leading to complex phenotypes.

### Methods

The computational model used here is EmbryoMaker, an overall description of the model is provided in S1 Text, a detailed description can be found in the original publication of EmbryoMaker[63]. In this article we consider several cell behaviors apoptosis, cell contraction and expansion (which can be asymmetric between the apical and basal side of epithelial cells), cell division, cell growth and extracellular matrix (ECM) secretion. Additionally, different cell mechanical properties are also included such as size, morphological plasticity (the plastic reduction of cell’s size due to external pressure), cell adhesion, resistance to compression and, for epithelial cells, resistance to epithelial bending. See S1 Text and S1T Text for a comprehensive explanation of the properties considered in this article. See Fig 1 and S1A Text for the complexity measures.

## 1. Ensemble approach: Building random networks

The ensemble approach is similar to that of a previous publication from our group[10]. All simulations started with 126 epithelial cells organized in a flat hexagonal sheet with an underlying layer of 126 mesenchymal cells (see Fig 2A). This morphology was chosen as the initial one because of its simplicity. The initial values of the mechanical properties were chosen so that if they remain unchanged, no morphological changes will occur. All simulations started with the same values in their mechanical properties (see S1 Text). Simulations were then numerically integrated using the 4^th^ order Runge-Kutta method with a dynamic step size. In order to halt the simulations, several stop conditions were implemented. Simulations had a limited number of iterations they could run for, which is approximately 3 physical days (see S1 Text). Additionally, simulations resulting in largely aberrant morphologies were discarded (e.g. consisting of broken epithelia, see S1 Text for a full description).

EmbryoMaker allows for rich gene regulation dynamics, but for simplicity these were reduced when building the developmental mechanisms for the ensemble. In this work, we do not distinguish between the different levels of gene expression (transcriptional, translational, etc.) and we consider that all cells express receptors for all the growth factors in a developmental mechanisms and do not consider signal transduction pathways in detail. Other modification to EmbryoMaker were implemented, for an exhaustive list of these changes see S1 Text.

Random developmental mechanisms were built by making random genes regulate the expression of random genes, random mechanical properties, or random cell behaviors. (Fig 2B). Each developmental mechanism is, thus, a gene network and the regulation of some cell behaviors or mechanical properties. Here, a set of interactions between a group of genes, which is organized by the genome, defines a gene network. Therefore, a gene network simply determines which genes interact if they are expressed in the same cell at the same time. To clarify, two gene products will not necessarily encounter each other, whether they do or not depends on the developmental dynamics (e.g. as run in EmbryoMaker).When constructing gene networks, genes were, with equal chance, either an intracellular gene product or an extracellularly-diffusible gene product (e.g. a growth factor). In every developmental mechanism, we chose that one gene product, gene product 1, directly activates an extracellularly diffusible gene product. All gene networks initially consisted of 10 genes, in which each gene had a 0.2 probability of regulating any gene in the network. Each regulation has a 0.5 chance of being either positive (activator) or negative (inhibitor) with a random regulatory strength between 0 and *t*_*max*_ (see S1 Text for a description of how is *t*_*max*_ determined) with uniform distribution. Therefore, on average, genes have two positive and two negatives connections (two efferent and two afferent).

To finish building the developmental mechanisms, cell behaviors and cell mechanical properties were added to the gene networks. Each gene in a gene network is given a 0.5 chance to regulate a cell mechanical property or a cell behavior (notice this regulation would only occur in those cells where such a gene becomes expressed during the simulations). To establish the value of this regulation, depending on the cell behavior being regulated, a random value along a logarithmic or a uniform distribution was chosen. (S1 Text). Additionally, the values of the degradation and diffusion rate of the gene product was also randomly chosen. Finally, all cells had a default small rate of cell division and differentiation since cell divisions occur in essentially every developing embryo.

In the first ensemble we tried, which we call the *broad ensemble* (S1 Text), the number of developmental mechanisms leading to complex morphologies was negligibly small. Because of this we build a simpler ensemble, the signaling-only ensemble (S1 Text and [10]), in which cells were not allowed to move, grow or divide, i.e. only cell signaling was allowed. In addition, gene one was expressed in a gradient across the initial morphology (Fig 2B). This ensemble allowed us to quickly find developmental mechanisms capable of pattern transformation. Specifically, we identified developmental mechanisms leading to temporally stable pattern transformations. Using these developmental mechanisms, we built the signaling ensemble (as in [10], see also S1 Text). In the results we refer to the signaling ensemble simply as the ensemble. In this ensemble, as in the broad ensemble, some of the genes in each developmental mechanism regulate some randomly chosen cell properties or cell behaviors. As a consequence, cells could move which allows for morphogenesis to occur. 20,000 developmental mechanisms were built for this ensemble.

## 2. Local exploration

To explore in detail the GPM and other properties of the developmental mechanisms in the ensemble, we chose 700 developmental mechanisms (which we call from now on the parental set, or simply, the parents) from the signaling ensemble. These 700 developmental mechanisms were chosen based on the complexity of the morphology they produce so that morphologies were evenly spread over a complexity range (from 0 to 1.2 AV). Notice that each developmental mechanism in the ensemble comes with a specific set of values in their developmental parameters and that these parameters, and their numbers, differ between developmental mechanisms. All developmental mechanisms in the parental set were simulated 10 times. Each resulting morphology we call the twin of a parental since it developed from exactly the same developmental mechanism. The twins may differ in morphology because of noise during development, which is implemented in EmbryoMaker as small random fluctuations added to cell positions over developmental time. The morphological distance between twins is the measure of developmental instability we use (see section 3).

Because the developmental mechanisms were built randomly, they contained many superfluous interactions that had no effect on development (i.e. no morphological change was observed when these interactions were deleted). We eliminated these superfluous interactions from further analyzes. Once the superfluous interactions were eliminated, we performed two kinds of local explorations on the parental set: a regular one-mutant neighborhood screening and an iso-morphological random walk.

### 2.1 Network pruning

To identify superfluous interactions in a developmental mechanism the following procedure was followed: 0) We took a parent from the parental set 1) A randomly chosen interaction was eliminated from the developmental mechanism producing it, lets call A the developmental mechanism that has such interaction and B the developmental mechanism were such interaction has been eliminated. 2) We simulated B 10 times and obtain 10 twin morphologies. 3) We calculate the average CMD (see 4.2) between the 10 twins of B and the 10 parental twins. If this average distance is higher than the developmental instability of the parent (using CMD) plus 0.01 the pruned morphology is considered different and is rejected. The 0.01 is empirically determined, anything above this threshold changes the morphologies in a noticeable way. This procedure was repeated (steps 1 to 3) until no deletion was accepted for 40 consecutive steps (95% of the parent networks had less than 40 interactions).

### 2.2 One-mutant neighborhood screening

In this screening we explore the morphological variation arising from small genetic variation of a parent. By small we mean variation affecting only a single parameter. Each offspring, thus, differs from its parent in only a parameter. We did a screening for IS-mutations, one for T-mutations involving deletions and T-mutations involving the addition of a new interaction in a gene network.

#### 2.2.1. Interaction-strength mutations (IS-mutations)

For each parameter in a parent we generated 8 mutant offspring. Each mutant consisted in modifying a parameter value proportionally to its parents value. Thus, -80%, -60%, -40%, -20%, +20%, +40%, +60% and +80% mutant offsprings were generated. The set of these mutants for each parent defines the one-mutant neighborhood of that parent. Note that each developmental mechanism may have different number of parameters, i.e. interactions, and then, the number of mutant offspring in the one-mutant neighborhood may differ between parents (i.e. there are 8 per parameter). Each mutant offspring was simulated ten times using different random seeds (10 twins per mutant).

#### 2.2.2. T-mutations:Deletion one-mutant neighborhoods

As for the IS-mutations, but by eliminating one interaction at a time.

#### 2.2.3. T-mutations: Addition one-mutant neighborhoods

As for the deletion one-neighborhood but adding a random interaction per mutant offspring (either between gene products or between a gene a cell behavior or cell mechanical property). These interactions are added in the same way than when constructing the signaling ensemble. For each developmental mechanism we generated *N*_*c*_ mutant offspring, where *N*_*c*_ is the number of interactions in the parent developmental mechanism. This way we ensured that roughly the same number of deletions and additions where essayed for each developmental mechanism (i.e. the number of possible deletions per developmental mechanisms is the number of interactions it has).

### 2.3. Iso-morphological random walks

We use these walks to estimate the size of the region of the parameter space in which a specific morphology can form. Note that each developmental mechanism can have a different number of parameters, which can result in the parameter space of different developmental mechanisms to have different dimensionality. In spite of that, we devise a way to estimate these sizes in a comparable way. The iso-morphological random walk follows a procedure similar to that pursued for pruning. Instead of eliminating interactions (i.e. T-mutation) we performed IS-mutations (i.e. changes in the parameters). These mutations were accepted if they lead to no morphological change and rejected if they did. The size of the iso-morphological region was then calculated as the proportion of rejected mutations in a random walk. In other words, the larger the proportion of mutations that change a phenotype, the smaller is the region of the parameter space in which a morphology forms.

The iso-morphological random walks were performed only in very stables parents (EMD distance between parental twins less than 0.3). We performed 10 random walks per developmental mechanisms, each walk with 200 steps (i.e. IS-mutations). Every mutation changed one parameter at a time. Each mutation changed a parameter by adding or subtracting two times the value of that parameter in the parent. To avoid that developmental noise could affect when a morphology is considered equal to the parent, each mutant was simulated 5 times (5 twins). The morphological distance between the 5 twins and the parental was measured using CMD (convexity morphological distance) and the average of these was used as the distance from the mutant to the parent. In order to be considered different to the parental, the CMD between mutants and parent had to be 0.01 higher than developmental instability of the parental (also measured in CMD). This threshold was determined visually by comparing 100 morphologies from a wide range of complexities. In the range of morphological complexity of the parents, a change of less than 0.01 CMD is hardly noticeable visually. In addition, our calculations are not much affected by noise, since we only used very stable morphologies (EMD < 0.3).

## 3. Morphological distances

### 3.1 Euclidean minimal distance (EMD)

This is a very convenient method to measure the distance between morphologies with different number of cells and does not require the use of landmarks of morphological features [52]. Therefore, this measure is especially useful for the embryos studied here, since they are often made of different number of cells and their morphologies can be quite different. EMD is the average Euclidean distance from a given node in a morphology to the closest node in another morphology. To measure this mean distance, first we measure the distance between each node in a morphology (morphology 1) to the closest node in the other morphology (morphology 2). Second, we repeat the previous step, but this time from morphology 2 to morphology 1. Finally, the sum of this distances between nodes is divided by the total number of nodes in morphology 1 and 2. The distance between morphology 1 and morphology 2 is then:

$$EMD=\frac{1}{n_{1}+n_{2}}\left(\sum_{k=1}^{n_{1}}\;d_{k,min(k,2)}+\sum_{j=1}^{n_{2}}\;d_{j,min(j,1)}\right)$$

Where *n*_*1*_ and *n*_*2*_ is the number of nodes in morphology 1 and 2 respectively, *d*_*k*,*min(k*,*2)*_ is the distance between node k in morphology 1 and its closest node in morphology 2, *d*_*j*,*min(j*,*1)*_ is the distance between node j in morphology 2 and its closest node in morphology 1. It is important to notice that the closet node from morphology 1 to morphology 2 is not necessarily the same that the other way around, i.e. the minimal distance relationship is not symmetric. EMD has the advantage that it can compare morphologies that differ in size and number of nodes. In addition, EMD can be calculated for any pair of morphologies without the need of establishing any correspondence or homology between points in the two morphologies being compared.

### 3.2 Homology between nodes

The way the ensemble is built allows establishing clear homologies between cells in two morphologies since all morphologies develop from the same initial morphology. Essentially, we label the cells in the initial condition and establish that cells in two different morphologies are homologous if they have the same label. When a cell divides in our simulations, one gets the label of its mother while the other gets a new label. Which daughter keeps the label is determined at random. Although cells move over simulation time, epithelial cells tend to maintain their neighbors. In addition, there is a limited number of divisions in our simulations, i.e. we start with 542 epithelial nodes and finish simulations when there are 5000 nodes. This relatively small number of divisions and the small change in neighbors ensures that all the offspring of a cell in the initial condition form a relatively closed clone. As a result, it does not make much difference which of the daughter cells receives the original label.

#### 3.2.1 Convexity morphological distance (CMD)

In this method we measure the local convexity of the epithelium around each epithelial node of a morphology and then compare it with that of each homologous node in another morphology. The local convexity around a node is measured as follows: The set of initial epithelial nodes is S = {1,2,3,…,542}. For each node in S we calculate the unit vector between node S_i_ and the other node in the same cell, this gives v_1_ (note all epithelial cells are made of an apical and a basal node). We then calculate the unit vectors between node S_i_ and each neighboring node in the plane of the epithelium (i.e. nodes touching node S_i_) which are of the same type (apical or basal). This give us a set of vectors V_i_. Next, we calculate the dot product between v_1_ and each vector in V_i_. The proxy for the local curvature of node *i* will be the average of these dot products. Irrespectively of the orientation of a morphology in 3D space the local convexity of a node is close to 0 if the neighboring nodes are in the same plane, 1 if the node is in a evagination of the epithelium and -1 if it is in an invagination (S1D Text). The convexity of a node is then:

$$l_{s_{i}}=\frac{1}{n_{i}}\sum_{k=1}^{n_{i}}\left(v_{1}\cdot v_{k}\right)$$

Where $l_{s_{i}}$ is local curvature of node i, n is number of elements in vector V_i_, v_1_ is unit vector 1 and v_k_ the is unit vector k in set V_i_. To obtain the distance between two morphologies, we calculate mean absolute difference in convexity between the homologous cells of two morphologies.

$$d_{1,2}=\frac{1}{542}\sum_{i=1}^{n_{i}}l_{1i}-l_{2i}\vee$$

Where l_1i_ is the local convexity of morphology 1 at node i, l_2i_ is the local convexity of morphology 2 at node i and 542 is the number of initial epithelial nodes.

#### 3.2.2 Homologous morphological distance (HMD)

For all the twins of a given combination of developmental parameters we calculate the mean morphology as the mean position of each homologous node. This helps to reduce the effect of developmental instability on morphology. The morphological distance between two mean morphologies is then calculated as the procrustes distance: the square root of the sum of the square of the differences between the positions of each pair of homologous nodes.

## 4. Complexity measures

We measure complexity based on how easy it is to predict the position of an epithelial cell if we know the 3D coordinates of its neighbors’ cells. For example, the position of a cell in a flat epithelium can be easily predicted by knowing the position of its neighbors, since they all share the same z-axis position. However, on a folded epithelium, especially if folded irregularly, it will be more difficult to predict the position of a cell based on the position of its neighbor cells. Therefore, epithelia folded in an irregular way, we consider very complex. To get an idea of how each complexity measure works and to see several examples of morphologies, see Figs 1 and 5. Based on this ideas, and to avoid that our results are bias by a single way of measuring complexity, we used two different ways of measuring complexity:: angle-distance variance and orientation patch count.

### 4.1 Angle-distance variance (AV)

For this measure we calculate the angle variation between different epithelial cells. To calculate the angle between two epithelial cells, we measure the angle between the apical-basal vectors of cell i and the apical-basal vector between cells i and j (see Fig 1A). The first step to calculate AV is to measure the angles calculated this way between cell i and all other epithelial cells. The second step is to classify the obtained angles in seven categories based on the distance between the cells and cell i (see Fig 1B). Each angle is assigned to one of seven distance intervals, which are defined as:

$$D_{c}=\{c\times p^{\overset{´}{ADD}},(c+1)+p^{\overset{´}{ADD}}\};c\in \{3,\ldots ,9\}$$

Where D_c_ is the distance interval to which cells are assigned depending to their distance to cell i, and that will determine to which category c they belong to. c defines the range of each interval and $p^{\overset{´}{ADD}}$ is the average distance of adhesion of all epithelial cells in the embryo. The smallest distance interval used is c = 3, in order to preclude noise from affecting the measurement. The different interval levels are used to consider small morphological structures (c = 3) all the way up to c = 9, which captures the macro-structure of the embryo. The third step is to calculate the variance of angles found in each category c and add them together. These steps are performed for each epithelial node. The final angle variation complexity (AV) will be:

$$AV=\frac{\sum_{i=1}^{n}\sum_{c=3}^{9}\;V_{ic}}{7n}$$

where i is each of the epithelial cells, n is the total number of epithelial cells in the embryo, c is each of the category intervals and V_ic_ is the angle variation for cell i in the category c. With this complexity measurement, a perfect sphere will have zero complexity.

### 4.2 Orientation patch count (OPC)

This complexity measure considers the number of patches in an epithelium that have different slope orientation. This method has been previously used to correlate teeth complexity to diet [11], although the version used here is applied to 3D embryos. The first step for this method, is to assign each epithelial cell to one of eight categories. This categories correspond to one octant (see S1A Text). In order to assign a cell to an octant, we calculated the vector between the apical and the basal side of each epithelial cell. Using the sign of each of the dimensions of this vector we stablish to which octant the cell belongs. The second step is to group cells into patches. A patch is a group of cells included in the same orientation category (in the same octant) and continuously connected to each other. Therefore, one can go from any given cell in a patch to any other cell in the same patch without changing the orientation category (S1A Text). In order to avoid inflating the number of patches due to noise, patches with less than four cells were not considered. The final OPC value results from adding the number of patches found in a morphology.

## 5. Genotype phenotype map (GPM) measures

To quantify the complexity of the genotype-phenotype map (GPM) we measure the regression coefficient (*β*) of the “genetic” distances (x-axis) versus the phenotypic distances of the mutants (y-axis) of each parent in the parental set. Each of these plots we call a “GPM regression plot” of a parameter and parent in the one-mutant neighborhood screening. In these plots (Fig 8A–8C) we also include the parental developmental mechanism without mutations. The genetic distance we plot in the x-axis. Each mutant diverges from the parent in only one parameter and at different proportions in respect to its value in the parent (from -80% to +80% in 20% steps). The genetic distance is this proportion. In the y-axis we plot the morphological distances, as we have three different ways of measuring morphological distances, we plot the “GPM regression plots” using each of these distances. When measuring the morphological distance with EMD and with CMD, each of the points in the scatter plot of the “GPM regression plots” indicates the average distance between all the twins of one mutant against all the twins of the other mutant. When using HMD as the morphological distance, each point indicates the distance between two average mutant morphologies. By following this procedure, we obtain plots as in Fig 8A–8C, where in the x-axis 0% position we have the distances between the twins of the mutants (i.e. their developmental instability), and for example, in the 160% position we have the phenotypic distance between the +80% mutant and the -80% mutant. Notice that for the HMD “GPM regression plots” there is no 0% genetic distance, as all the twins are used to obtain a single average morphology.

With this method, when small genetic changes lead to big changes in the phenotype, the regression coefficient will be high, and we consider the GPM to be complex. Notice also that morphologies with higher developmental instability do not necessarily have higher regression coefficients, as we can see in Fig 8B and 8C. Even though C has a higher developmental instability than B, as the morphological distances between the different mutants does not change with their genetic distance, they both have small regression coefficients. The regression coefficient is, thus, a measure of the GPM that is not affected by developmental instability. Having such a property is important because complex morphologies in the ensemble are more developmentally unstable than simple morphologies.

## 6. Degeneracy

Degeneracy occurs when similar morphologies can be achieved by more than one developmental mechanism. To observe to which degree simple and complex morphologies are degenerated we measured the morphological distance (using HMD) between the morphologies arising from the developmental mechanisms in a random subset of developmental mechanisms in the signaling ensemble. If degeneracy is high among morphologies of a given complexity, these should exhibit short morphological distances between them. Developmental mechanisms are chosen based on whether they produce morphologies evenly distributed across certain complexity intervals, 0 to 1.2 in 0.1 steps for AV and 0 to 25 in 2 steps for OPC. For each complexity step we found 20 morphologies. We then plotted the average morphological distance between the different complexity intervals. This results in a heatmap were the distances between the different complexity intervals can be observed.

## Supporting information

S1 Text Supporting information for “On the evolution and development of morphological complexity: a view from gene regulatory networks”. (PDF)
