# Correction: On the evolution and development of morphological complexity: A view from gene regulatory networks

**DOI:** 10.1371/journal.pcbi.1009686

**Published:** 2021-12-10

**Authors:** Pascal F Hagolani, Roland Zimm, Renske Vroomans, Isaac Salazar-Ciudad

There are errors in the funding statement. The correct funding statement is as follows:

This study has been funded by the Spanish Ministry of Science PGC2018-096802-B-I00 (https://www.ciencia.gob.es/) and the Academy of Finland Centre of Excellence in Experimental and Computational Biology (Suomen Akatemia) AA123456 (https://www.aka.fi/en/). The funders had no role in study design, data collection and analysis, decision to publish, or preparation of the manuscript.
